# Drug Design and Delivery for Intracellular Bacteria: Emerging Paradigms

**DOI:** 10.1002/ddr.70198

**Published:** 2025-11-19

**Authors:** Babatunde Ibrahim Olowu, Maryam Ebunoluwa Zakariya, Abdulmuheez Abiola Abdulkareem, Olalekan Toheeb Okewale, Muhammad Halima Idris, Halimah Oluwayemisi Olayiwola

**Affiliations:** ^1^ Multidisciplinary Program in Infectious Diseases, Department of Veterinary Microbiology, College of Veterinary Medicine Washington State University Pullman Washington USA; ^2^ Program in Global Health and Infectious Diseases, Edinburgh Medical School, College of Veterinary Medicine The University of Edinburgh Edinburgh UK; ^3^ School of Pharmacy, Faculty of Health Sciences University of Eastern Finland Kuopio Finland; ^4^ Department of Veterinary Pharmacology and Toxicology, Faculty of Veterinary Medicine University of Ibadan Ibadan Nigeria; ^5^ Department of Veterinary Pharmacology and Toxicology, Faculty of Veterinary Medicine University of Abuja Abuja Nigeria; ^6^ Department of Pharmacy, Faculty of Pharmacy University of Ibadan Ibadan Nigeria

**Keywords:** biomimetic therapeutics, host‐directed therapy, intracellular bacterial pathogens, pharmacokinetics/pharmacodynamics (PK/PD), subcellular targeting, targeted drug delivery

## Abstract

Intracellular bacteria exploit host cell niches, such as lysosomes, phagosomes, cytosol, entire cells, and even erythrocytes, to evade immune clearance and escape conventional antibiotics. These environments pose numerous therapeutic challenges, including crossing host cell membranes, navigating endosomal trafficking, tolerating acidic and redox conditions, bypassing efflux mechanisms, and countering phenotypic tolerance. Although recent advancements in nanotechnology—such as carriers, prodrugs, and host‐directed therapies—offer promising solutions, current strategies remain narrowly focused on “getting the drug inside the cell”, leaving therapeutic agents vulnerable to off‐site targeting, degradation, and functional failure. This review introduces a next‐generation approach for intracellular antibacterial therapy, incorporating subcellular targeting, dual‐function delivery systems, innovative biomimetic carriers, precise intracellular pharmacokinetics/pharmacodynamics (PK/PD) assessment, and artificial intelligence‐assisted drug design. Highlighting frameworks for multimodal regimens targeting intracellular bacteria, we advocate a transition from solely facilitating cellular entry to achieving precise spatiotemporal regulation of drug activity within infected host cells. This paradigm informs the development of therapeutics designed to persist within the intracellular bacterial niche, minimizing relapse and reducing the emergence of antimicrobial resistance.

**Abbreviations**
ActA: actin assembly‐inducing protein a (*Listeria monocytogenes* surface protein mediating actin polymerization and intracellular motility)
ADMET: absorption, distribution, metabolism, excretion, and toxicity
AI: artificial intelligence
AMPs: antimicrobial peptides
APC: antigen‐presenting cell
ATP: adenosine triphosphate
BCV: Brucella‐containing vacuole
CNN: convolutional neural network
DL: deep learning
DOT/Icm: defective organelle trafficking/intracellular multiplication system
EB/RB: elementary body/reticulate body (Chlamydia life cycle forms)
ER: endoplasmic reticulum
IBP: intracellular bacterial pathogen
LC3: microtubule‐associated protein 1A/1B‐light chain 3 (autophagy marker)
LPS: lipopolysaccharide
LSTM: long short‐term memory (neural network architecture)
MDR: multidrug resistance
MDT: multidrug therapy
ML: machine learning
MLR: multiple linear regression
NN: neural network
NPs: nanoparticles
OVM: outer membrane vesicle/outer vesicular membrane
PEG: polyethylene glycol
PEGylated: chemically modified with polyethylene glycol
PK/PD: pharmacokinetic/pharmacodynamic
PLSR: partial least squares regression
PNP: polymeric nanoparticle
QSAR: quantitative structure–activity relationship
RF: random forest (machine learning model)
RND: resistance–nodulation–division (efflux pump family)
RNS: reactive nitrogen species
ROS: reactive oxygen species
SCV: small‐colony variant (*Staphylococcus aureus* persistent phenotype)
SVM: support vector machine
T3SS: type III secretion system
T4SS: type IV secretion system
Vd: volume of distribution virtual ligand screening

## 1 Introduction

Intracellular bacterial pathogens continue to pose a significant global health challenge, not only due to their capacity for antimicrobial resistance but also because they utilize host cells as safe havens (Salam et al. 2023). Pathogens such as *Mycobacterium tuberculosis* (Sankar and Mishra 2023), *Salmonella enterica* (Leoni Swart and Hensel 2012), *Listeria monocytogenes* (Nowacki et al. 2025), *Chlamydia trachomatis* (Murray and McKay 2021), and facultative intracellular strains of *Staphylococcus aureus* exploit host cells, especially macrophages, dendritic cells, and epithelial cells, as reservoirs (Lathram and Radka 2025). In addition, *S. aureus* is known to invade osteoblasts, establishing intracellular sanctuaries that contribute to chronic osteomyelitis. Within these niches, intracellular bacterial pathogens evade immune responses, survive for long periods, and are shielded from many antimicrobials that cannot enter, act within, or be effective inside the relevant intracellular compartments (Kamaruzzaman et al. 2017).

Numerous obstacles hinder the effectiveness of intracellular bacterial (Subramaniam et al. 2021), primarily, challenges related to host cell entry and trafficking is most paramount because many super‐therapeutics antibiotics that are being formulated have limited ability to cross mammalian cell membranes and/or are hijacked into endo‐lysosomal compartments, where they are subjected to degradation and/or fail to reach bacteria inside modified phagosomes, the cytosol, or vacuoles with altered pH and redox conditions (Chen et al. 2023). Intracellular retention and stability also pose significant challenges (Chan and Tsourkas 2024), as drugs may be expelled through efflux mechanisms (Gaurav et al. 2023), broken down by cellular or lysosomal enzymes, sequestered, or rendered inactive by intracellular factors such as acidity and oxidative stress (Zhong et al. 2024). This is in complement to the underlying challenges of antibacterial targets within bacteria, like the cell wall, ribosomes, and DNA gyrase, which need to be accessible after overcoming both the host cell and bacterial cell envelope barriers, requiring penetration of both barriers (Kamaruzzaman et al. 2017).

A more translational challenge is the discrepancies between pharmacokinetic and pharmacodynamic (PK/PD) parameters, which present a significant challenge (Zhang et al. 2022). It is frequently observed that systemic plasma concentrations, tissue levels, or extracellular metrics often exhibit weak correlations with drug concentrations within bacterial cells, thereby impacting therapeutic efficacy (Alikhani et al. 2025). Hence, the adoption of conventional dosing protocols, which, even though they achieve high serum levels, may not ensure or maintain sufficient intracellular exposure, contributing to treatment failure, disease relapse, latency, and the emergence of antibiotic resistance (Karsdal et al. 2025).

Over the years, nanotechnological advancements have led to frontiers in combating these challenges (Tripathi et al. 2025; J. H. Tian, Huang et al. 2025). Potent tools such as biomimetic and stimuli‐responsive nanomaterials provide controlled release, enhanced cellular uptake, and targeted subcellular delivery (Tripathi et al. 2025; Yang et al. 2025). These investigations have demonstrated that the size, shape, ligand conjugation, and carrier composition of pharmacological agents critically determine their behavior, cellular trafficking, and susceptibility to lysosomal degradation (Chen et al. 2023; J. H. Tian, Huang et al. 2025). Nevertheless, the precise intracellular localization, infection stage, and strain‐specific virulence variations of intracellular bacterial pathogens are often not comprehensively understood, resulting in a persistent knowledge gap (Thakur et al. 2019; Lazar et al. 2023; van Schaik et al. 2024).

There is a need for a better understanding of how intracellular bacterial pathogens remodel host trafficking, manipulate membrane compartments, and reprogram metabolism to create permissive niches, for insight into future catalysts of pharmacotherapeutics and drug‐delivery system engineering (Mahieu et al. 2025; Phat et al. 2025). Hence, this review presents a structured framework for the next generation of intracellular antibacterial therapies. By correlating known pathogenic functions to notable exploitable strategies, we aim to catalyze a paradigm shift, from merely achieving cellular entry to precisely engaging pathogens at their intracellular sites of residence, bridging pharmacology, nanomedicine, and systems biology.

## 2 Niches and Barriers to Therapy

Intracellular bacterial pathogens inhabit a diverse array of host cell types, each providing a unique environment that influences pathogenesis and therapeutic efficacy (Thakur et al. 2019; Table 1). Comprehending the correlative state and functions of these niches is fundamental to the development of therapies capable of accurately targeting and acting at the infection site.

**Table 1:** Intracellular bacterial pathogens, their niches, and therapeutic implications.

Pathogen	Intracellular niche	Pathogen dynamics	Therapeutic implications	References
*Mycobacterium tuberculosis*	Arrested phagosome	Inhibiting phagolysosome fusion; tolerates acidic and nitrosative stress	Acid‐stable drugs; host‐directed therapies restoring phagosome maturation	Chandra et al. (2022)
*Mycobacterium leprae*	Schwann cell phagosomes	Hijacks Schwann cell pathways; causes demyelination	Requires prolonged MDT; nerve‐targeted therapies	Avanzi et al. (2020)
*Salmonella enterica*	Salmonella‐containing vacuole and cytosol	SPI‐2–regulated vacuolar/cytosolic lifestyle; adapts to nutritional stress	Dual‐release carriers responsive to pH/ROS (vacuole + cytosol)	Q. Li (2022)
*Listeria monocytogenes*	Cytosol	Escapes vacuole; replicates freely in cytosol; triggers immune activation	Cytosol‐active antibiotics; enhance cell penetration	X. Li et al. (2025)
*Shigella flexneri*	Cytosol	Exploits actin polymerization to spread cell‐to‐cell	Cytosolic growth inhibitors; block actin motility	Schnupf and Sansonetti (2019)
*Chlamydia trachomatis*	Inclusion vacuole	Avoids immune detection; supports EB/RB biphasic cycle	Inclusion‐penetrant prodrugs or peptide conjugates	Elwell et al. (2016)
*Chlamydia pneumoniae*	Inclusion vacuole	Respiratory pathogen with chronic/persistent forms	Prolonged macrolide/tetracycline regimens	Elwell et al. (2016)
*Coxiella burnetii*	Acidified parasitophorous vacuole	Thrives in acidic lysosome‐like niche	Acid‐stable drugs; proteolysis‐resistant compounds	van Schaik et al. (2024)
*Brucella abortus*/*melitensis*	ER‐derived vacuole (BCV)	Converts phagosome into ER‐like replicative niche	ER‐targeted nanocarriers; adjuncts immunotherapy	de Figueiredo et al. (2015)
*Francisella tularensis*	Cytosol (after transient phagosome)	Highly cytotoxic; intracellular replication precedes dissemination	Rapid cytosolic release systems + immune stimulation	Ozanic et al. (2015)
*Legionella pneumophila*	ER‐like Legionella‐containing vacuole	Hijacks host vesicle trafficking via Dot/Icm effectors	ER‐targeted delivery; inhibitors of Dot/Icm effectors	Escoll et al. (2017)
*Rickettsia rickettsii*	Cytosol with actin‐based motility	Escapes vacuole; uses actin tails to disseminate	Fast‐acting cytosolic antibiotics; immune boosting	Huang et al. (2022)
*Rickettsia prowazekii*	Cytosol	Strict cytosolic growth; minimal metabolic flexibility	Cytosolic targeting; immune clearance enhancement	Valbuena and Walker (2009)
*Anaplasma phagocytophilum*	Apoptosis‐resistant vacuole (morulae)	Blocks apoptosis; reprograms neutrophil genes	Neutrophil‐permeant antibiotics; block morula formation	Truchan et al. (2016)
*Ehrlichia chaffeensis*	Monocyte/macrophage morulae	Modulates monocyte signaling, immune suppression	Monocyte vacuole‐penetrant drugs; immunomodulation	McBride and Walker (2011)
*Orientia tsutsugamushi*	Cytosol	Rapid phagosome escape; cytosolic persistence	Fast cytosolic release drugs, vaccine adjuncts	Richards and Jiang (2020)
*Bartonella henselae*	Endothelial cell vacuoles	Induces angiogenesis	Endothelium‐targeting drugs, antiangiogenic approaches	Harms and Dehio (2012)
*Bacillus anthracis (spores)*	Macrophage phagosome	Spore germination before systemic spread	Macrophage‐penetrant early therapy to block dissemination	Guidi‐Rontani et al. (2001)
*Yersinia pestis*	Early macrophage phagosome	Facultative intracellular, transient vacuole survival	Early‐phase intracellular coverage critical	Perry and Fetherston (1997)
*Neisseria gonorrhoeae*	Epithelial vacuoles; transcytosis	Invades epithelia; transcytoses to submucosa	Mucosal‐penetrant antibiotics; epithelial transcytosis inhibitors	Quillin and Seifert (2018)
*Neisseria meningitidis*	Endothelial vacuoles	Crosses BBB using transcytosis	BBB‐permeable drugs required	Stephens et al. (2007)
*Helicobacter pylori(intracellular forms)*	Gastric epithelial vacuoles	Occasional intracellular niche, contributes to persistence	Intracellular‐active gastric delivery systems	Necchi et al. (2017)
*Burkholderia pseudomallei*	Cytosol	Escapes vacuole, uses actin‐based motility	Cytosolic targeting and actin motility blockade	Allwood et al. (2011)
*Burkholderia mallei*	Cytosol	Like *B. pseudomallei*	Cytosolic delivery systems	Syed and Wooten (2021)
*Rhodococcus equi*	Arrested phagosome	Inhibits phagosome‐lysosome fusion	Phagosome‐active antibiotics with acid stability	Prescott (1991)
*Wolbachia spp*.	Cytoplasmic vacuole	Symbiont of insects; obligate intracellular	Not a therapeutic target in humans (model only)	Werren et al. (2008)
*Treponema pallidum*	Possible transient intracellular stage	Mostly extracellular but can transiently enter host cells	Extracellular‐targeted therapy usually sufficient	Radolf et al. (2016)
*Ureaplasma urealyticum*	Host cell cytoplasm	Evidence for cytoplasmic persistence under stress	Experimental intracellular delivery possible	J. Song et al. (2022)
*Clostridioides difficile (spores)*	Macrophage phagosomes	Spores resist killing inside phagosomes	Macrophage‐active germination inhibitors	Paredes‐Sabja et al. (2014)
*Staphylococcus aureus (SCVs)*	Endosomes, phagosomes, cytosol	Persists as SCVs, evades immune clearance, contributes to chronic infection	Vacuole escape strategies + SCV‐targeted antibiotics	Gunn et al. (2024)

### 2.1 Host Cell Types

#### 2.1.1 Macrophages

Macrophages constitute one of the most extensively studied niches (Herb et al. 2024); *M. tuberculosis* persists within macrophages by modulating host endocytic trafficking and inhibiting phagosome–lysosome fusion, thereby avoiding exposure to degradative hydrolases and reactive oxygen intermediates (Kellermann et al. 2021; Maphasa et al. 2021). This arrested phagosomal environment enables the pathogen to survive for prolonged periods in a semidormant state. Similarly, *S. enterica* targets macrophages but remodels its phagosome into the *Salmonella*‐containing vacuole (SCV) (Steele‐Mortimer 2008), establishing a specialized compartment with a distinct ionic and protein composition that facilitates bacterial replication while minimizing detection by immune sensors (Leoni Swart and Hensel 2012).

#### 2.1.2 Dendritic Cells

Dendritic cells function not only as immune sentinels but also as intracellular sanctuaries that certain pathogens utilize for survival and dissemination (Leoni Swart and Hensel 2012). *S. enterica* can persist within human and murine dendritic cells (Bueno et al. 2008; Leoni Swart and Hensel 2012). Additionally, *Salmonella* disrupts antimicrobial pathways and alters host transcriptional programs to evade antigen presentation and subsequent interactions with cytotoxic T cells, mediated by dendritic cells (Leoni Swart and Hensel 2012; Jensen et al. 2016; Aulicino et al. 2022). Dual RNA‐seq analyses indicate that *Salmonella* actively reconfigures dendritic cell metabolism and immune signaling to enhance its survival (Aulicino et al. 2022). This reprogramming facilitates the trafficking of dendritic cells to lymphoid tissues, enabling systemic dissemination (Avital et al. 2017; Aulicino et al. 2022). Similarly, *L. monocytogenes* is internalized by dendritic cells and can persist intracellularly for a sufficient duration to initiate immune evasion, a process mediated by CD8α⁺ dendritic cell subsets, which have been identified as playing a critical role in early pathogenesis (Eldridge and Hamon 2021).

#### 2.1.3 Epithelial and Endothelial Cells

Epithelial and endothelial cells form a vital interface for interactions between the host and pathogens, functioning as significant reservoirs for intracellular bacteria, especially at mucosal and vascular barriers (Zhou et al. 2025). These cells not only serve as entry points but also establish protective niches that safeguard pathogens from circulating immune effectors (Caven et al. 2023). *Chlamydia trachomatis* develops a highly specialized membrane‐bound inclusion within epithelial cells, hijacking host trafficking machinery to intercept Golgi‐derived exocytic vesicles, redirect sphingolipids and cholesterol, and prevent lysosomal fusion (Murray and McKay 2021; Caven et al. 2023). Additionally, the pathogen manipulates host cytoskeletal networks and type III secretion systems to reposition the inclusion near the microtubule‐organizing center, thereby enhancing access to host resources and evading autophagy detection (Colonne et al. 2016).

#### 2.1.4 Hematopoietic Cells

Hematopoietic cells constitute some of the most formidable niches; *Anaplasma phagocytophilum* selectively infects neutrophils, persisting within endosome‐like inclusions, where it inhibits NADPH oxidase assembly, thereby preventing oxidative killing (Eskeland et al. 2023). This pathogen also utilizes type IV secretion effectors, such as AFAP, which interact with nucleolin to suppress apoptosis (D. Zhang et al. 2025), and it exploits ATG14‐ and ULK1‐dependent autophagy pathways to facilitate intracellular replication (Turck et al. 2025). *Ehrlichia chaffeensis* inhabits monocyte‐derived vacuoles, employing Etf‐1 to inhibit apoptosis and leveraging Notch signaling to upregulate XIAP, thereby prolonging host cell survival (Lin et al. 2023; Patterson et al. 2023). Certain species also infect erythrocytes, which are anucleate cells lacking endocytic and lysosomal pathways, rendering them immune‐privileged and markedly resistant to pharmacological clearance (Clemente et al. 2023).

### 2.2 Subcellular Compartments

In addition to cell types, intracellular bacterial pathogens inhabit distinct subcellular niches, each requiring unique pharmacological considerations (Kellermann et al. 2021).

#### 2.2.1 Phagosomes

Phagosomes function as the initial environment for various bacteria; however, multiple pathogens inhibit the maturation and acidification of phagosomes. For instance, *M. tuberculosis* impedes the fusion of phagosomes with lysosomes, whereas *S. enterica* extensively alters its phagosome, transforming it into a replication‐permissive *Salmonella*‐containing vacuole (SCV) (Steele‐Mortimer 2008; Kellermann et al. 2021).

#### 2.2.2 Phagolysosomes

Certain pathogens thrive within acidic phagolysosomes, as demonstrated by *Coxiella burnetii*, which survives within a large, acidified vacuole abundant in endo‐lysosomal and autophagic markers (M. Zhao et al. 2024). This compartment, designated as the *Coxiella*‐containing vacuole (CCV), constitutes a stable and permissive environment for replication (Wan et al. 2024).

#### 2.2.3 Specialized Vacuoles or Inclusion Bodies

Other pathogens avoid lysosomal targeting entirely by establishing specialized vacuoles or inclusion bodies. *Chlamydia trachomatis* resides in a nonacidic inclusion that intercepts Golgi‐derived vesicles, acquires host sphingolipids, and circumvents fusion with degradative compartments (Murray and McKay 2021). Similarly, *A. phagocytophilum* and *E. chaffeensis* replicate within morulae—unique vacuole‐like inclusions that remain nonfusogenic with lysosomes and are selectively enriched for nutrients required for bacterial growth (Clemente et al. 2023).

#### 2.2.4 ER‐Associated Vacuoles

ER‐associated vacuoles represent an additional niche. *Brucella abortus* modifies its vacuole into an endoplasmic reticulum (ER)‐derived compartment, known as the *Brucella*‐containing vacuole (BCV), which acquires ER markers and provides a protected environment conducive to persistent infection (Celli 2019). Certain pathogens manipulate autophagy‐related pathways to facilitate intracellular survival (Mulcahy et al. 2020); *A. phagocytophilum* recruits autophagy proteins such as Beclin‐1 and ATG5 to its inclusion, thereby redirecting autophagosomes as a source of nutrients, whereas *Shigella flexneri* actively inhibits autophagic capture by secreting effectors that obstruct LC3 conjugation or by promoting actin‐based motility to evade immune responses (Ogawa et al. 2005; Niu et al. 2012).

#### 2.2.5 Cytosol

Several pathogens, including *L. monocytogenes, Shigella*, and many *Rickettsia* species, are able to completely escape into the cytosol. Residency within the cytosol allows access to host metabolites, yet it also exposes these pathogens to cytosolic immune surveillance (Pizarro‐Cerdá and Cossart 2018). *Listeria* uses ActA to recruit the Arp2/3 complex, facilitating cell‐to‐cell spread without exiting into the extracellular environment. This strategy facilitates evasion of immune detection and circumvention of antibiotics with limited cellular penetration (Pizarro‐Cerdá and Cossart 2018).

### 2.3 Barriers to Therapy

#### 2.3.1 Membrane Uptake, Trafficking, pH, and Redox

Many antibiotics are too hydrophilic or bulky to freely diffuse across host cell membranes, relying instead on endocytosis, which can lead to entrapment in endo‐lysosomal compartments where drugs are degraded or isolated from pathogens (Subramaniam et al. 2021). Intracellular trafficking can direct therapeutics into compartments that do not overlap with pathogen niches, thereby reducing their efficacy. Phagosomal and lysosomal acidity (pH 4.5–5.0) can destabilize acid‐labile drugs, protonate weak bases, causing sequestration, or alter drug activity. Reactive oxygen and nitrogen species further stress bacteria and can chemically modify drugs, while forcing pathogens into metabolically quiescent states that are less susceptible to antibiotic killing (Thakur et al. 2019).

#### 2.3.2 Efflux, Sequestration, Persistence

Efflux transporters in host cells and bacteria actively expel antibiotics, diminishing intracellular concentrations. Bacterial efflux pumps, including RND‐family transporters, are upregulated in response to intracellular stress, thereby extruding key drug classes (Gaurav et al. 2023). Hydrophobic antibiotics may partition into lipid droplets or bind to intracellular proteins, thereby lowering their bioavailability (Subramaniam et al. 2021). Moreover, phenotypic heterogeneity generates subpopulations of persisters with transient metabolic shutdown, enabling survival even at bactericidal drug levels and contributing to relapse after therapy (Thakur et al. 2019).

## 3 Principle of Small Molecule Design

The rational design of small molecules for intracellular bacterial pathogens must integrate classical pharmacokinetic principles with the biological heterogeneity of drug exposure across organs, tissues, cell types, subcellular compartments, and even individual bacteria (Fan and de Lannoy 2014; Day et al. 2024). Pharmacokinetic descriptors—absorption, distribution, metabolism, and excretion (ADME)—remain essential but are insufficient to explain the variability seen in intracellular infection models (Abel Zur Wiesch et al. 2017). Drug exposure is not homogeneous: local tissue concentrations, cellular metabolic activity, transporter expression, and subcellular distribution fluctuate dynamically and frequently decouple from systemic plasma levels (C. Wang et al. 2023). A deeper understanding of these multiscale determinants is critical to ensure that candidate molecules achieve adequate exposure at their precise intracellular sites of action.

### 3.1 Physicochemical Descriptors of Intracellular Penetration

Among the most informative molecular descriptors for predicting intracellular penetration are lipophilicity, polarity, and ionization state (Mugumbate and Overington 2015; Piccaro et al. 2015; Wardecki et al. 2023). These parameters are summarized in Table 2, which links optimal ranges with permeability outcomes and therapeutic implications (Table 2).

**Table 2:** Physicochemical descriptors of intracellular pharmacological fate for bacterial pathogens.

Descriptor	Optimal range/property	Effect on permeability and intracellular fate	Therapeutic implications	References
Lipophilicity (logP/logD)	0–3 (neutral compounds). logP > 5 = excessive tissue sequestration	Drives passive membrane diffusion; very high lipophilicity can cause intracellular sequestration and reduce free drug levels	Balances oral absorption and distribution; most marketed drugs fall within 0–5; macrolides achieve high intracellular exposure due to moderate lipophilicity	Piccaro et al. (2015); Pea (2018); Wardecki et al. (2023)
Polar surface area (PSA)	< 60 Å^2^ → > 90% absorption; 60–140 Å^2^ = moderate permeability; > 140 Å^2^ = poor passive uptake	High PSA impedes passive diffusion; often requires active transporters	β‐lactams, aminoglycosides (PSA > 140 Å^2^) poorly penetrate host cells and remain extracellular, limiting efficacy vs. intracellular pathogens	Mugumbate and Overington (2015); Qiu et al. (2019)
pKa (Ionization State)	Weakly basic (6–8) for balanced cytosolic diffusion and lysosomal trapping	Determines charge state; affects permeability and subcellular distribution	Lysosomotropic drugs (pKa > 6, logP ~2–6) accumulate in acidic compartments; can prolong half‐life but risk tissue‐specific toxicity	Yousef et al. (2023)
Molecular weight	< 500 Da preferred (Lipinski′s Rule of 5)	Smaller molecules diffuse efficiently across membranes; favorable for intracellular penetration	Classical small‐molecule drugs with reliable oral bioavailability and broad tissue distribution	Lipinski (2004)
Molecular weight	> 500–2000 Da (macromolecular or conjugated drugs)	Larger molecules cross membranes more slowly; diffusion depends on transporters or carrier systems	Characteristic of peptide conjugates, macrolides, and nanoparticle‐linked drugs optimized for intracellular delivery	Chen et al. (2022)
Transporter interactions	Substrate for uptake transporters (OATP2B1, PHT1/2)	Uptake transporters increase intracellular exposure; reduced by efflux pumps	Activity is restored by efflux inhibitors, and it improves intracellular retention	Rautio et al. (2018); A. Sharma et al. (2019)
Prodrug strategies	Enzyme‐activated, pH‐triggered, redox‐activated	Improve permeability, bypass efflux, and achieve compartment‐specific release	Isoniazid (KatG‐activated) and ethionamide (EthA‐activated) exemplify pathogen‐specific bioactivation	Hanoulle et al. (2006); Huttunen et al. (2011); Xavier et al. (2021)

#### 3.1.1 Lipophilicity

Lipophilicity, expressed as logP (or logD for ionizable compounds), governs passive membrane diffusion and tissue partitioning (Wardecki et al. 2023). Compounds with moderate lipophilicity (logP between 0 and 3) generally achieve the best balance between permeability and aqueous solubility, whereas highly lipophilic drugs (logP > 5) tend to accumulate in lipid‐rich tissues, lowering the concentration of freely available drug at the target site (Mugumbate and Overington 2015; Piccaro et al. 2015). The volume of distribution (Vd) is often used as a surrogate for cellular partitioning, with a high Vd suggesting intracellular sequestration (Pea et al. 2005; Pea 2018).

#### 3.1.2 Polarity

Polarity, typically measured as polar surface area (PSA), also critically affects intracellular access (Mugumbate and Overington 2015). Passive diffusion across membranes is generally favored when PSA values are below 140 Å^2^, with near‐complete absorption observed for PSA < 60 Å^2^, whereas highly polar antibiotics such as β‐lactams and aminoglycosides (logP < 0, PSA > 140 Å^2^) require active transport systems and often fail to reach therapeutic intracellular concentrations ((Ertl 2007; Qiu et al. 2019; Tang et al. 2023; Thy et al. 2023).

#### 3.1.3 Ionization State

The ionization constant (pKa) further modulates membrane permeability and lysosomal trapping. Basic drugs (pKa > 6, logP~2–6) readily cross membranes in their un‐ionized form but become protonated and trapped in the acidic environment of lysosomes (pH~5), resulting in intracellular accumulation that can enhance efficacy but also predispose to tissue‐specific toxicity (De Duve et al. 1974; Derendorf 2020; Yousef et al. 2023).

#### 3.1.4 Molecular Weight and Interaction

Molecular weight impacts diffusion rates: compounds < 500 Da typically cross membranes more efficiently and exhibit favorable oral bioavailability (Lipinski 2004). Transporter interactions add another layer of complexity: uptake transporters (OATP2B1, PHT1/2) can enhance intracellular exposure, whereas efflux pumps (e.g., MRPs) reduce drug retention (Mateus et al. 2017; Rautio et al. 2018; A. Sharma et al. 2019)

### 3.2 Transporter‐Mediated Uptake, Efflux, and Intracellular Retention

Even with optimized physicochemical properties, intracellular drug concentrations can be undermined by efflux and metabolism. Macrophages express uptake transporters, such as PHT1, PHT2, and OATP2B1, which facilitate entry; however, they also express efflux pumps, including multidrug resistance proteins (MRPs), that lower intracellular exposure (Mateus et al. 2017; Rautio et al. 2018). Intracellular bacterial pathogens likewise express resistance‐nodulation‐cell division (RND)‐family efflux pumps that actively extrude antibiotics, compounding the problem (Moreau et al. 2011; A. Sharma et al. 2019; Vergalli et al. 2020). Co‐administration with efflux pump inhibitors (EPIs) represents a rational co‐therapeutic strategy to restore antibiotic activity and prolong intracellular retention (Sharma et al. 2019).

### 3.3 Prodrug Design

When the physicochemical properties of a lead compound cannot be optimized to achieve sufficient permeability, stability, and potency simultaneously, prodrug strategies become valuable (Testa 2009; Huttunen et al. 2011; Jubeh et al. 2020; Weng et al. 2022). Carrier‐linked, bioprecursor, and double prodrugs are engineered to improve solubility, membrane permeability, and activation at the site of infection. Several frontline antitubercular agents are themselves prodrugs: isoniazid is activated by the mycobacterial catalase–peroxidase KatG, ethionamide by the monooxygenase EthA, and pyrazinamide by bacterial amidase (Hanoulle et al. 2006). pH‐activated prodrugs exploit the acidic environment of endosomes and lysosomes for selective release, an approach first described in early PEGlytated conjugates (Simplício et al. 2007) and further advanced in recent polymeric nanocarrier designs (Song et al. 2019; Chu et al. 2022; Luo et al. 2023; Junyaprasert and Thummarati 2023) and PEGylated PLGA systems (Dereje et al. 2025) support the continued innovation in pH‐responsive delivery. Redox‐activated prodrugs such as metronidazole require reduction under anaerobic conditions to generate reactive intermediates that damage bacterial DNA, RNA, and protein targets; recent work has highlighted the role of mycobacterial nitro‐reductases such as Mrx2 (Rv2466c) in activating nitroaromatic antibacterials (Jubeh et al. 2020; Xavier et al. 2021; Subramaniam et al. 2024; Eke and Abramovitch 2025).

### 3.4 Trojan‐Horse and Targeted Uptake

“Trojan‐horse” strategies exploit host or bacterial nutrient uptake systems to enhance intracellular access. Siderophore–antibiotic conjugates (SACs) hijack bacterial iron transport pathways, while classic sideromycins such as ferrimycin, salmycin, and albomycin demonstrate receptor‐mediated uptake and potent activity against Gram‐negative pathogens (Górska et al. 2014; Dassonville‐Klimpt and Sonnet 2020; Rayner et al. 2023; Gräff and Barry 2024). Nanoparticle (NP) carriers offer another Trojan‐horse approach, as they are readily phagocytosed by host macrophages, facilitating intracellular antibiotic delivery; their size, surface charge, and chemistry can be tuned to direct trafficking toward pathogen‐containing compartments (Dassonville‐Klimpt and Sonnet 2020) Carbohydrate‐modified cyclodextrins and glycoconjugate antibiotics further exploit glucose transporters (GLUTs), which are upregulated in metabolically active infected cells, to achieve preferential uptake and intracellular accumulation, resulting in up to 100‐fold reductions in MICs for macrolides, rifamycins, and fluoroquinolones (M. Li et al. 2016; Matović et al. 2020).

### 3.5 Phenotypic Versus Target‐Based Discovery

Ultimately, discovery strategies must be tailored to the unique requirements of intracellular efficacy. Phenotypic screening in macrophage infection models provides the most direct evidence of activity, as it captures permeability, efflux, stability, and potency simultaneously, including effects on nonreplicating or tolerant bacterial subpopulations (Khare et al. 2013; Aulner et al. 2019; Ellis et al. 2019; Melander et al. 2022; Vincent et al. 2022) However, it does not initially reveal the molecular target, which complicates optimization and toxicity prediction (Pries et al. 2016). Target‐based discovery allows rapid structure–activity relationship (SAR) development but is limited by poor correlation between enzyme inhibition and whole‐cell efficacy, particularly when host barriers or efflux prevent intracellular target engagement (Vasaikar et al. 2016; Croston 2017; S. Ghosh and O'Connor 2017; Compagne et al. 2023; X. M. Pei et al. 2023; V. Kumar et al. 2024). Ideally, modern pipelines integrate both approaches, using target‐based methods for hit finding and phenotypic models for validation under physiologically relevant conditions.

## 4 Drug Delivery Platforms

Molecular optimization alone is inadequate for ensuring sufficient drug concentrations within infected host cells; therefore, drug delivery platforms serve as essential mechanisms to overcome physiological barriers (Chifiriuc et al. 2016; Sánchez et al. 2020). These systems can protect antibiotics from degradation, enhancing bioavailability and promoting accumulation at the infection site (Mukherjee et al. 2019; Chen et al. 2023; Table 3).

**Table 3:** Drug delivery platforms: Mechanisms, advantages, limitations, and design roles in intracellular antibacterial therapy.

Delivery platform	Mechanism	Pros	Cons	Drug design role	Key references
Liposomes	Adsorption, fusion, encapsulation, or conjugation of drug within a lipid bilayer vesicle; PEGylation prolongs circulation; allows passive (EPR) and ligand‐mediated targeting	Enhances therapeutic index, protects labile drugs, overcomes enzymatic degradation	Encapsulation instability, chemical degradation, leakage (especially oral due to pH), scale‐up and batch variability.	Enhances macrophage uptake, provides sustained drug release, and enables ligand‐directed targeting of infected tissues or immune cells	Narang et al. (2013); He et al. (2019); R. Ghosh and De (2023)
Polymeric nanoparticles (PNPs)	Biodegradable polymer matrices (PLGA, PEG‐PLGA) encapsulate or adsorb drugs, enabling controlled or stimuli‐responsive release	Biodegradable, tunable size and release profile, good drug stability, broad cargo compatibility	Protein corona formation, opsonization, physiological barriers, and manufacturing complexity.	Used for sustained release of hydrophobic antibiotics, improved intracellular retention, and co‐delivery of multiple agents (e.g., antibiotics + immunomodulators).	Sánchez et al. (2020)
Lipid–polymer hybrids (LPHNs/PLNs)	Polymeric core provides drug reservoir and stability, lipid shell improves colloidal stability, reduces leakage, and supports surface functionalization	Combines liposome biocompatibility with polymer stability, reduces premature drug loss, supports sustained release	Aggregation risk, physiological characterization challenges, more complex fabrication.	Combines stability of NPs with targeting potential of liposomes; suitable for mannose‐ or antibody‐conjugated macrophage‐targeted therapies	Jiang et al. (2020); Shah et al. (2022)
Dendrimers (e.g., PAMAM)	Branched, tree‐like macromolecules allowing internal encapsulation and surface conjugation of drugs; can be stimulus‐responsive	High multivalency, biofilm inhibition, can carry multiple agents simultaneously	High cost, cytotoxicity (esp. cationic generations), clearance challenges.	Useful for multivalent drug delivery, co‐loading of efflux pump inhibitors, and targeted delivery via surface functionalization	Pryor et al. (2014); Ezeh and Dibua (2024)
ryoNanogels	Cross‐linked hydrophilic polymer networks that swell in response to pH, enzymes, or temperature, enabling controlled release and barrier crossing	High drug loading capacity, injectable, mimic soft tissue, potential for dermal/transmucosal delivery	Reproducibility and stability concerns, burst release possible.	Enables pH‐triggered drug release in acidic phagolysosomes and targeted toxin neutralization using RBC‐nanogels	Rajput et al. (2020); Vashist et al. (2024)
Cell‐penetrating peptides (CPPs)	Short cationic/amphipathic peptides conjugated to cargo; enter cells via direct penetration or endocytosis, releasing drug into cytoplasm	Broad cargo range (proteins, nucleic acids, drugs), modular and easy to synthesize	Endosomal entrapment, off‐target uptake, limited membrane translocation in vivo.	Facilitate cytosolic delivery of hydrophilic antibiotics or nucleic acids; can be engineered for stimuli‐responsive activation in infected tissue	Klipp et al. (2023)
Antibody–antibiotic conjugates (AACs)	Antibody selectively binds bacteria; cleavable linker releases antibiotic intracellularly after internalization	Precision targeting, reduces systemic antibiotic exposure, effective against intracellular reservoirs	Complex manufacturing, immunogenicity, pharmacokinetic risks.	Delivers antibiotics directly to bacteria within host cells, reducing off‐target exposure and resistance pressure	Lehar et al. (2015)
Engineered vesicles/exosomes	Naturally secreted vesicles engineered to carry siRNA, CRISPR, or small molecules; surface modified for targeting	Cross biological barriers, low immunogenicity, inherent tropism	Yield heterogeneity, cargo loading challenges, regulatory complexity.	Provide biomimetic delivery with potential for cell‐specific targeting and immune evasion	Luan et al. (2017); Elsharkasy et al. (2020)
Phage‐based targeted delivery	Phages surface‐modified or used as nanocarriers for drug or CRISPR cargo; exploit bacterial tropism and biofilm penetration	High specificity, can overcome antimicrobial resistance, synergize with antibiotics	Rapid clearance by immune system, storage stability, regulatory complexity.	Used for precision elimination of MDR bacteria or delivery of gene‐editing agents inside intracellular pathogens	M. Zhao et al. (2023); Marchi et al. (2025)
Yeast‐based delivery (β‐glucan particles)	Hollow yeast shells or β‐glucan particles loaded with drug; recognized by dectin‐1 on macrophages/DCs, directing intracellular delivery	Safe, orally bioavailable or inhalable, good protection of cargo, targets innate immune cells	Variability in yeast cell prep, need for size/purity control, immunostimulant risk.	Ideal for macrophage‐targeted delivery of anti‐TB drugs or immunostimulants	Upadhyay et al. (2017)
Antimicrobial peptides (AMPs)	Disrupt bacterial membranes via pore formation, inhibit nucleic acid or protein synthesis, and may modulate host immunity	Multiple mechanisms, low risk of resistance emergence	Cytotoxicity potential, reduced activity in physiological saline.	Serve as direct antimicrobials or as conjugates to enhance intracellular drug uptake and activity	Le et al. (2017); Cresti et al. (2024)

### 4.1 Conventional Platforms

#### 4.1.1 Polymers Nanoparticles (NPs)

Polymeric NPs serve as matrix‐integrated capsules, protecting the drug cargo, enhancing its stability, and facilitating controlled biodegradation (Lu and Huang 2020; Zhao et al. 2021; Hosseini et al. 2022). Recent studies have further optimized biodegradable and stimuli‐responsive polymers for intracellular drug delivery, improving macrophage targeting, endosomal escape, and pharmacokinetic profiles (Luo et al. 2023; Dereje et al. 2025). Notably, several studies in the context of mycobacterial therapy have shown that isoniazid‐conjugated NPs or clofazimine (CFZ)‐loaded NPs significantly enhance bacterial clearance in vitro (Batalha et al. 2019; Hosseini et al. 2022). Nevertheless, concerns remain regarding their potential to trigger immune activation and cytotoxic effects, underscoring the importance of ongoing biocompatibility optimization.

#### 4.1.2 Dendrimers

Dendrimers are highly branched, water‐soluble macromolecules whose architecture radiates outward from a central core (Winnicka et al. 2013; Wrońska et al. 2015). Poly(amidoamine) (PAMAM) dendrimers have been explored as solubilizing agents to enhance the aqueous solubility of erythromycin, although this did not result in a significant improvement in antibacterial potency (Winnicka et al. 2013). More promising results have been observed in chlamydia treatment, where dendrimer‐based azithromycin carriers allowed more efficient and sustained intracellular drug delivery (Mishra et al. 2011; Wrońska et al. 2015). That said, cytotoxicity remains a major limitation, with severe toxicity documented for PAMAM dendrimers of sixth generation and above (Pryor et al. 2014).

#### 4.1.3 Lipid–Polymer Hybrids (LPH)

Lipid–polymer hybrid nanoparticles (LPHNs) combine a biodegradable polymeric core with a stabilizing lipid shell, effectively merging the strengths of both carrier systems (Mukherjee et al. 2019). Recent work has demonstrated their value in tackling methicillin‐resistant *S. aureus* (MRSA), where mannose‐modified PLGA cores loaded with vancomycin were encapsulated within a dual‐functional lipid layer to improve targeting and therapeutic response (Cai et al. 2025). The lipid coating not only enhances biocompatibility and colloidal stability but also helps limit premature drug leakage, while the polymeric core supports sustained drug release, creating a genuinely synergistic delivery platform for antibacterial therapy (Cai et al. 2025). That said, problems such as NP aggregation and the complexity of physiological characterization continue to hinder reproducibility and large‐scale translation, highlighting a key area for future optimization (Jiang et al. 2020).

#### 4.1.4 Liposomes

Liposomes are spherical vesicles made up of one or more lipid bilayers surrounding an aqueous core (Chifiriuc et al. 2016). They have shown therapeutic utility across multiple intracellular infection models—for example, meropenem‐loaded liposomes have been evaluated in Pseudomonas aeruginosa infections, while gentamicin encapsulation has been tested for Brucella therapy (Zahra et al. 2017). Their bilayer architecture enables the simultaneous loading of hydrophilic drugs in the core and hydrophobic agents within the lipid membrane, providing protection from degradation and allowing for ligand‐mediated targeting when surface‐functionalized (Zahra et al. 2017). Despite these advantages, challenges persist, including chemical instability, drug leakage, and susceptibility to denaturation, particularly within gastrointestinal environments or during prolonged storage, which continue to limit their clinical robustness (Narang et al. 2013). Consequently, current research focuses on improving lipid composition, PEGylation strategies, and scalable manufacturing processes to enhance stability and facilitate clinical translation (Huang et al. 2022).

#### 4.1.5 Nanogels

Nanogels are nanoscale, highly hydrated, cross‐linked polymer networks that can encapsulate therapeutic molecules while remaining responsive to environmental cues, such as pH, temperature, or enzymatic activity. Their soft, tissue‐like consistency allows them to penetrate infection sites and release their pharmacological agent in a controlled fashion. In antimicrobial applications, nanogels have been shown to disrupt bacterial membranes, dismantle biofilms, and inhibit key processes, including protein and DNA synthesis (R. Ghosh and De 2023). Beyond direct antibacterial action, they can be engineered for injectable formulations, mucosal delivery, or transdermal application, making them versatile tools for intracellular drug delivery (Y. Zhang et al. 2017). However, issues such as burst release, reproducibility of synthesis, and stability during storage remain important challenges for translation to clinical use (Huang et al. 2022).

### 4.2 Biologic Vectors

#### 4.2.1 Antimicrobial Peptides (AMPs) and Cell‐Penetrating Peptides (CPPs)

AMP and CPP serve as delivery vehicles for antibacterials or act directly on bacteria intracellularly. In testing *L. monocytogenes*, Arnett et al. (2011) used “α‐human defensins and RC‐1 humanized θ‐defensin, retrocyclin‐1.” Through non‐lytic mechanisms, only a few AMPs manage to reach intracellular bacteria (L. Yang et al. 2020). CPPs have been covalently conjugated with gentamicin to target Gram‐negative bacteria, including *S. flexneri* (Gomarasca et al. 2017). However, CPPs pose a challenge because, following treatment, intracellular bacteria remain trapped in endosomal compartments (Buccini et al. 2021).

#### 4.2.2 Phage and Yeast Targeted Drug Delivery

Phage‐targeted drug delivery can facilitate the sharing of pathogen burden between the host cell and the pathogen, as demonstrated in the study by Schmalstig et al. (2024), which focused on the use of phages to eliminate *M. abscessus*. However, phages are limited because, without specific measures, the immune system frequently clears them, thereby reducing the efficacy of the drug (H. Wang et al. 2024). Yeast cells contain porous β‐glucan particles with hollow cavities that are advantageous for facilitating therapeutic molecular adsorption and encapsulation. These particles have been loaded with Rifabutin (RB) to target macrophages for tuberculosis treatment, with a brief window (~5 min) observed before the phagocytosis of the particles by macrophages (Upadhyay et al. 2017).

## 5 Next‐Generation Strategies: Perspective Piece

### 5.1 Intracellular PK/PD

Intracellular bacterial pathogens pose a unique pharmacological challenge that undermines therapeutic effectiveness and clinical outcomes (Ngwalero et al. 2021). Agents with excellent systemic pharmacokinetics often perform poorly against pathogens sequestered within cellular compartments because plasma PK does not reliably predict intracellular exposure—particularly in vacuoles, phagosomes, and cytosolic niches where bacteria evade immune and antimicrobial responses. Recognizing and quantifying this disparity is essential, not merely as an academic exercise, but as the foundation for developing next‐generation precision delivery strategies (Mahieu et al. 2025).

#### 5.1.1 Why Plasma PK ≠ Intracellular Efficacy: Case Studies

**Figure 1:**
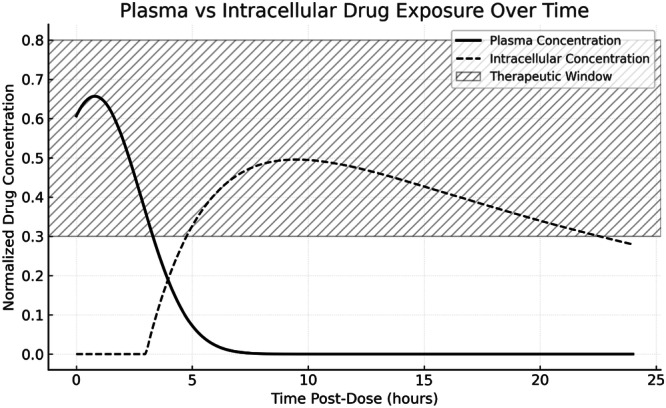
Schematic representation of plasma versus intracellular drug exposure over time. Plasma drug concentrations typically peak at an early stage and decrease rapidly, while intracellular concentrations tend to accumulate following a lag phase. The shaded area represents the therapeutic window (T > MIC) required for effective bacterial eradication (concept adapted from Carryn et al. 2002; Aljayyoussi et al. 2017; Ngwalero et al. 2021; Mahieu et al. 2025.

A key example comes from the study of “Bedaquiline” in *M. tuberculosis* (Ngwalero et al. 2021). In patients with drug‐resistant TB, (Ngwalero et al. 2021) demonstrated that plasma levels of bedaquiline and its active metabolite M2 do not always correlate with their proportional accumulation in patient peripheral blood mononuclear cells (PBMCs), an intracellular reservoir that can affect the drug's optimal efficacy (Ngwalero et al. 2021). Similarly, (Mahieu et al. 2025) validated an intracellular PD model using a hollow fiber infection system, demonstrating that intracellular bacterial pathogens generally exhibit a slower therapeutic response than their extracellular counterparts. This discrepancy arises from several interconnected factors, including restricted antibiotic penetration across host‐cell membranes, reduced intracellular drug concentrations within acidic or enzyme‐rich compartments, and the induction of metabolically quiescent or persister states that reduce the efficacy of growth‐dependent antimicrobials. Furthermore, intracellular localization affords protection from immune‐mediated clearance, collectively delaying bactericidal effects (Stokes et al. 2019; Liu et al. 2020; Lu et al. 2025). Consequently, therapeutic response may lag even when plasma pharmacokinetic/pharmacodynamic indices indicate sufficient systemic exposure (Mahieu et al. 2025). High‐content imaging of anti‐TB drugs supports this observation, visualizing delayed intracellular killing relative to plasma‐derived predictions (Aljayyoussi et al. 2017; Figure 1).

Together, these case studies emphasize the need to integrate extracellular and intracellular PK/PD data better to correlate pharmacological exposure with clinical bacterial load reduction. They also illustrate two key determinants of kill dynamics: (i) delays in intracellular exposure caused by pH‐dependent ionization, sequestration, or transporter effects relative to plasma concentrations, and (ii) differences in bacterial growth rates and metabolic states within host cells compared to conventional in vitro conditions (Aljayyoussi et al. 2017; Ngwalero et al. 2021; Mahieu et al. 2025).

#### 5.1.2 Measuring Intracellular PK/PD

##### 5.1.2.1 Live‐Cell Imaging

Fluorescently tagged antibiotics or biosensor reporters enable real‐time visualization of drug uptake, trafficking, and retention within host cells (Tantama et al. 2011). Combining fluoroscopy with advanced microscopy techniques—including confocal, spinning‐disk, and light‐sheet microscopy—provides detailed spatial resolution, allowing for the distinction between drug localization in the cytosol and endosomes/lysosomes (Tantama et al. 2011; Ma et al. 2017). Multispectral imaging enables the simultaneous tracking of multiple fluorescent probes, allowing for the observation of endosomal receptor trafficking with minimal phototoxicity and excellent temporal resolution (A. Kumar et al. 2025).

##### 5.1.2.2 Multimodal Quantification Framework

Multimodal quantification framework: Measuring drug exposure within the pathogen's subcellular niche requires integrating experimental and computational methodologies (Ma et al. 2017). Subcellular fractionation, combined with LC–MS/MS, enables the precise quantification of drug levels in the cytosol, vesicular compartments, and membranes, generating detailed intracellular concentration‐time profiles (Ma et al. 2017). These measurements can be paired with hollow fiber infection models to collect temporally resolved intracellular bacterial burden data under controlled drug exposures, allowing direct correlation between exposure and bacterial kill kinetics (Aljayyoussi et al. 2017; Donnellan et al. 2019; Mahieu et al. 2025)

#### 5.1.3 Relevant PK/PD Indices and Modeling for Intracellular Pathogens

PK/PD indices such as AUC/MIC, Cmax/MIC, and time above MIC (T > MIC) are standard tools for optimizing antibacterial therapy (Alikhani et al. 2025). However, these parameters are usually derived from plasma or extracellular measurements and therefore do not fully capture the pharmacodynamics occurring within intracellular reservoirs (Alikhani et al. 2025). Because the microenvironment inside macrophages, dendritic cells, and epithelial reservoirs can significantly alter drug exposure, compartment‐specific PK/PD metrics are necessary (Aljayyoussi et al. 2017; Thakur et al. 2019; Herbst et al. 2022).

Evaluating compartment‐specific AUC/MIC involves integrating cytosolic or vacuolar concentrations over time and normalizing them to the MIC of the intracellular pathogen (Khoei et al. 2019). Studies of M. tuberculosis have shown that intracellular exposure to bedaquiline lags plasma exposure and may not achieve bactericidal thresholds, even when the plasma AUC/MIC appears sufficient (Ngwalero et al. 2021). This finding highlights that plasma‐based targets can overestimate therapeutic efficacy, reinforcing the need for direct intracellular measurements (Ngwalero et al. 2021).

T > MIC, the duration during which compartmental drug levels exceed MIC, is especially critical for pathogens with slow growth or dormancy (e.g., *M. tuberculosis*, *B. abortus*) where prolonged exposure is required for sterilizing activity (Aljayyoussi et al. 2017; Mahieu et al. 2025). Compartment‐resolved concentration–time curves (cytosol vs. lysosome) offer additional insights, helping identify rate‐limiting steps such as endosomal trapping, delayed endosomal escape, or rapid efflux (Carryn et al. 2002; D. Pei and Buyanova 2019). Intracellular kill‐curve modeling, when integrated with these data, enables the generation of classical PK/PD parameters specific to intracellular pathogens (Aljayyoussi et al. 2017).

### 5.2 Strategic Precision Delivery

The physiological, immunological, and pharmacological barriers posed by intracellular bacterial pathogens demand a transition toward precision therapeutics (A. K. Sharma and Khuller 2001; Armenia et al. 2022). Incorporating intracellular PK/PD parameters into therapeutic engineering is crucial for designing next‐generation interventions that can synchronize spatial and temporal drug exposure. This approach supports subcellularly targeted release, host immune modulation, and biomimetic evasion of immune clearance (Armenia et al. 2022; Rao et al. 2022).

#### 5.2.1 Precision Subcellular Targeting

**Figure 2:**
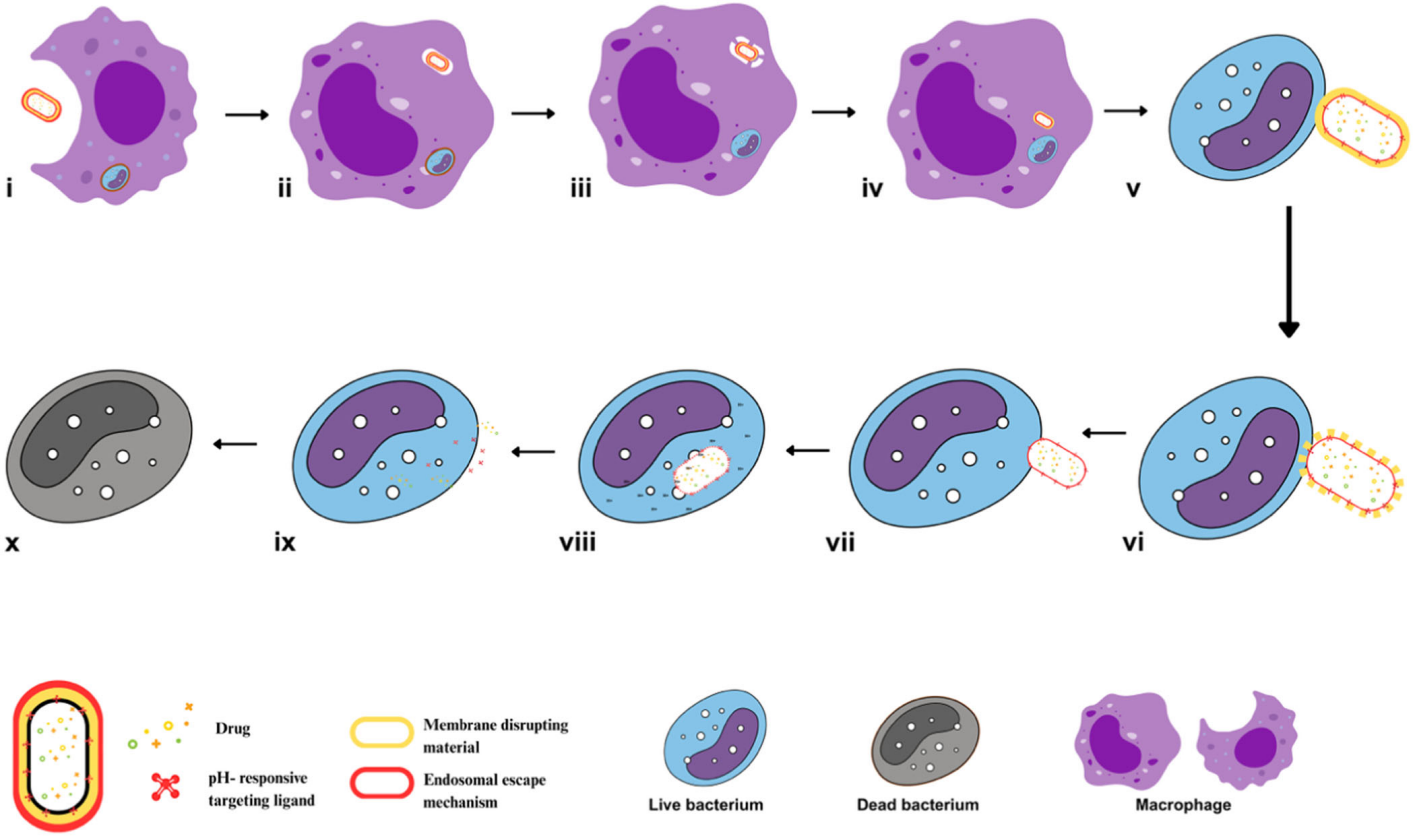
Schematic representation of precision subcellular targeting. (i–iv): Drug‐loaded nanoparticles or exosomes decorated with pH‐responsive targeting ligands and endosomal escape moieties are recognized and engulfed by macrophages through phagocytosis, leading to endosomal sequestration. (v): Magnified view of the boxed region in (iv), illustrating the subsequent trafficking of the carrier through the macrophage toward a bacterium‐infected host cell. (vi): Ligand–receptor interactions enable selective binding to the infected cell or pathogen. (vii–viii): The acidic endosomal environment activates membrane‐disruptive components, aiding in endosomal escape and the release of the therapeutic agent into the cytosol. (ix–x): The local drug action eradicates the intracellular bacterium, leaving a dead pathogen behind and preventing further infection.

The first step toward a successful eradication of IBPs is to match the drug delivery mechanics to precisely identify and reach the pathogen niche intracellularly (Omotade and Roy 2019; Figure 2). Stimuli‐responsive drug delivery systems (DDSs) exploit infection‐specific cues, pH shifts in phagosomes, redox gradients in inflamed tissues, and pathogen‐induced enzymatic activities to trigger site‐specific release of antibiotics and immunomodulators (Guo et al. 2018; Verkhovskii et al. 2023).

For instance, thioketal‐linked prodrugs remain inert in healthy tissues but are cleaved in reactive oxygen species (ROS)‐rich macrophages, resulting in site‐specific activation (Rao et al. 2022). A persistent barrier to efficacy is the sequestration of nanocarriers within late endosomes or lysosomes, where their pharmacological agent may be degraded or inactivated. To overcome this, next‐generation platforms incorporate endosomal escape mechanisms, including pH‐sensitive fusogenic lipids, membrane‐active peptides such as HA2 and TAT, and charge‐reversal polymers. These approaches destabilize endosomal membranes, enabling the release of cargo into the cytosol or pathogen‐modified vacuoles (Fortuni et al. 2019; Desai et al. 2024).

#### 5.2.2 Multiresponsive and Dual‐Trigger Systems

**Figure 3:**
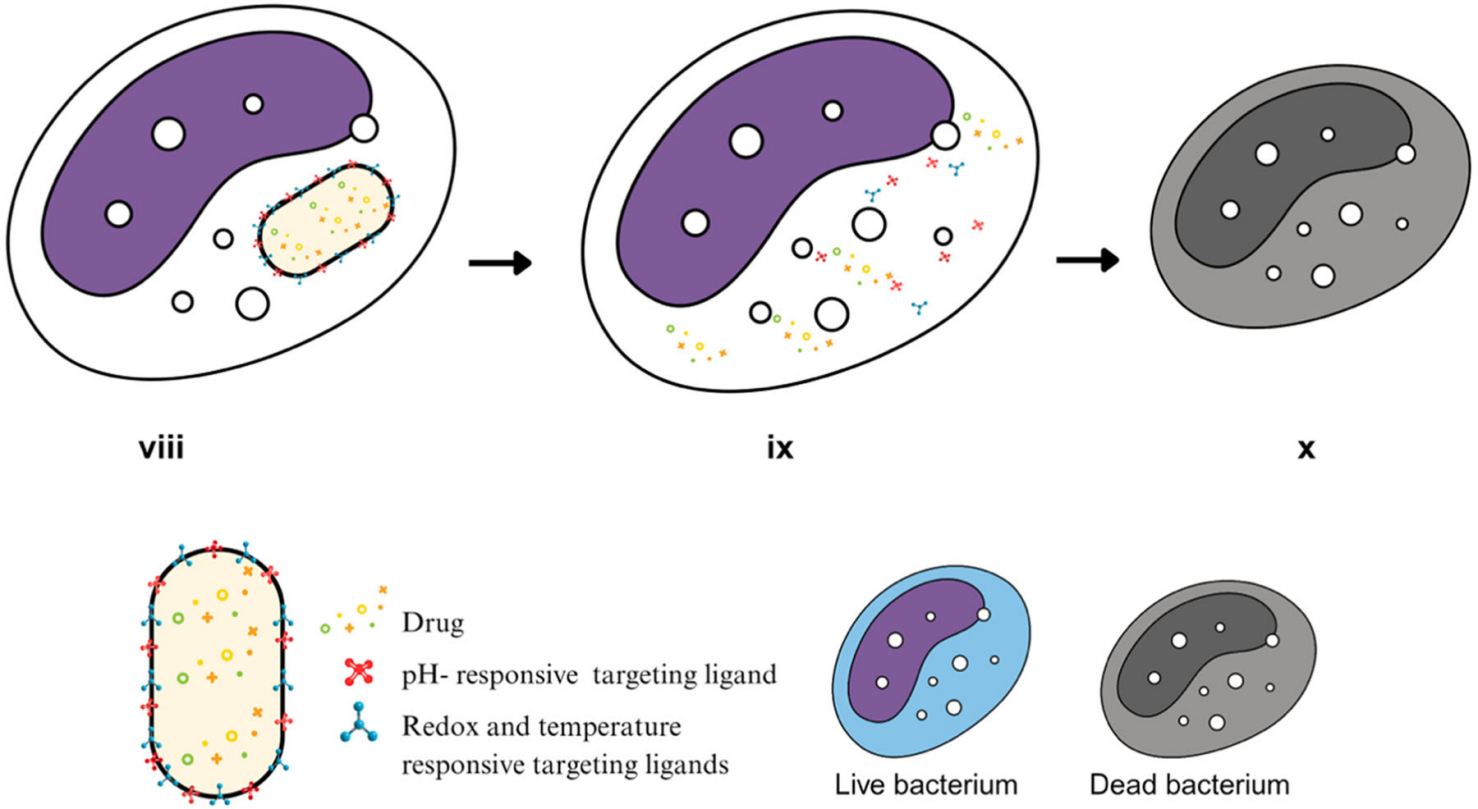
Multistimulus nanoplatform enabling precision release. (viii): Multifunctional nanocarrier decorated with pH‐, redox‐, and temperature‐responsive ligands, arrives at the pathogen‐containing compartment. (ix): Infection‐specific cues trigger coordinated drug release, assisted by endosomal escape and membrane destabilization, resulting in high local antibiotic concentration. (x): Intracellular pathogen is eradicated, leaving a cleared and functionally restored host cell.

Recent advances have moved beyond single‐trigger carriers toward dual‐ or tri‐responsive nanoplatforms that respond to combinations of stimuli such as ROS, glutathione, and acidic pH (Rao et al. 2022; Figure 3). These systems provide finely tuned release kinetics that closely mimic the microenvironments of infections. Poly(vinyl caprolactam)‐based nanogels containing disulphide linkages, for example, selectively degrade under redox and temperature conditions, releasing drugs in proximity to bacterial replication sites and concentrating their effects in the relevant microenvironment (Ling et al. 2019; Zhu et al. 2024). This strategy enhances spatial and temporal precision, minimizing systemic toxicity and counteracting bacterial immune evasion mechanisms (X. Li et al. 2025).

#### 5.2.3 Microenvironment‐Responsive Targeting

ROS production is a cornerstone of innate immune defence against pathogens such as *M. tuberculosis*, *S. enterica*, *L. monocytogenes*, and *S. aureus* (Murray and McKay 2021). Linkers responsive to ROS or pH, such as thioketal and boronate esters, allow nanocarriers to remain quiescent in healthy tissue and release their cargo only in inflamed or infected sites (J. Li et al. 2023; Alikhani et al. 2025). Integrating multiple triggers increases robustness, which is especially important for infections where pathogens actively suppress ROS generation (Alikhani et al. 2025).

#### 5.2.4 Pathogen‐Mimetic and Biomimetic Platforms

**Figure 4:**
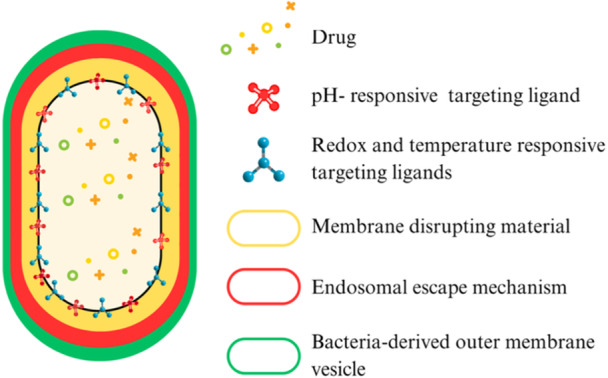
Schematics of a pathogen‐mimetic pharmacological nanocarrier.

The utilization of pathogen vesicle‐coated NPs, synthetic carriers enveloped in outer membrane vesicles (OMVs) derived from bacteria or their analogues, leverages the intrinsic tropism of OMVs for infected tissues and immune cells (Figure 4). These vesicles facilitate homing to bacterial colonization sites and enable the direct delivery of therapeutics into pathogen‐containing compartments (Liu et al. 2019; Fazal and Lee 2021). OMV‐functionalized mesoporous silica nanoparticles (MSNs) have demonstrated the ability to release antibiotics selectively upon exposure to bacterial enzymes or acidic pH levels, thereby ensuring targeted therapeutic action precisely where it is required (Colilla and Vallet‐Regí 2020; Fang et al. 2023). The integration of such coatings with orthogonal triggers, such as reactive oxygen species (ROS)‐cleavable or pH‐sensitive linkers, paves the way for the development of multilayered, infection‐responsive delivery systems distinguished by high spatial and temporal accuracy, heralding the future direction of targeted antimicrobial therapy (X. Li et al. 2025).

#### 5.2.5 Host‐Directed Therapy (HDT) and Dual‐Function Multisystemic Target

HDT aims to reprogram immune responses to enhance bacterial clearance while preventing excessive inflammation (Mishra et al. 2011). Combining HDT agents with bactericidal drugs amplifies autophagy, improves phagosomal acidification, and enhances ROS/RNS production, enabling macrophages to clear infection more effectively (Kaufmann et al. 2018; N. Tian, Chu et al. 2025; Tao et al. 2025). Mesenchymal stem cell–derived exosomes (MSC‐Exos) serve as natural carriers for antibiotics and immunomodulators, delivering them directly to infection sites while simultaneously polarizing macrophages toward a bactericidal phenotype and mitigating cytokine storm–associated damage (Mishra et al. 2011). This integrated approach offers a blueprint for complete pathogen sterilization.

#### 5.2.6 Novel Biomimetic Cloaking and Immune Camouflage

**Figure 5:**
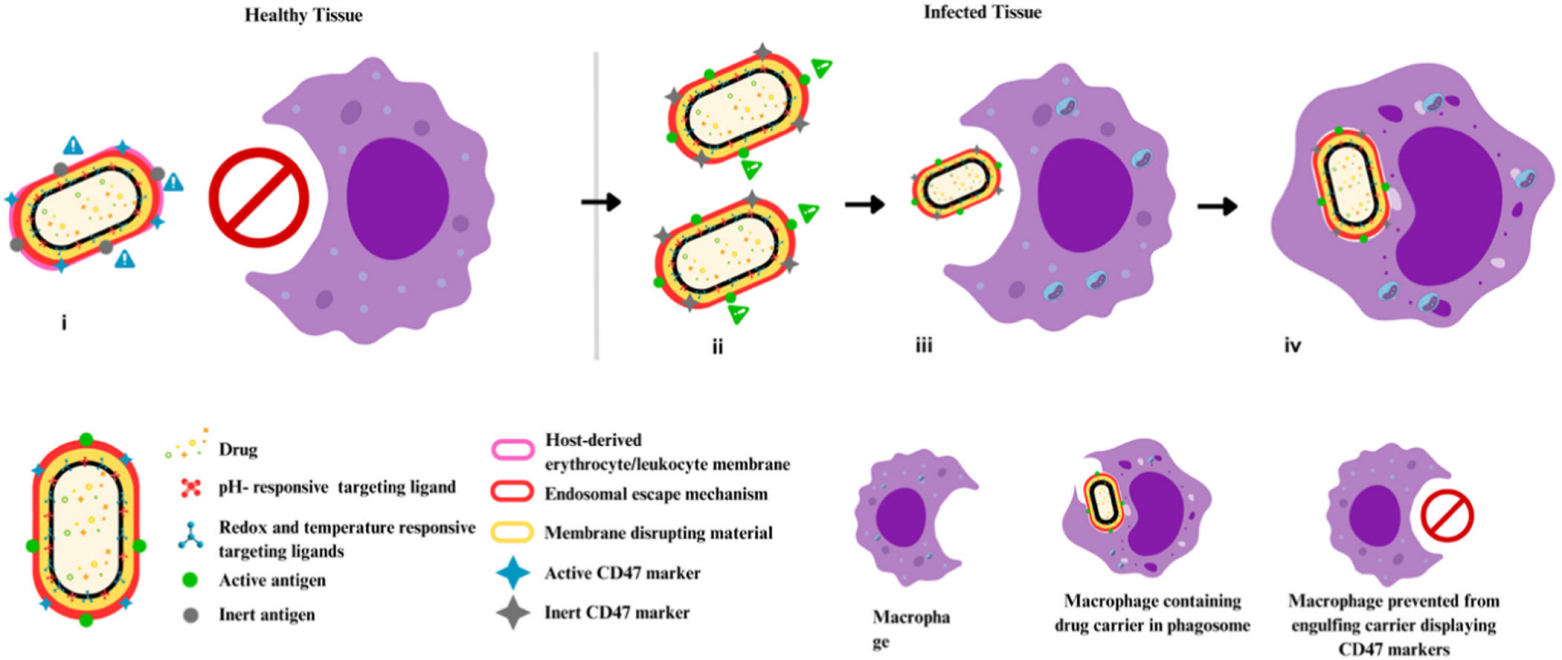
Schematics of dual‐biomimetic nanocarrier strategies. (Left, i): In healthy tissue, a host‐mimetic nanocarrier cloaked with erythrocyte/leukocyte‐derived membranes and expressing CD47 “self” markers avoids macrophage phagocytosis, thereby extending systemic circulation and decreasing off‐target clearance. (Right, ii–iv): In infected tissue, the pathogen‐mimetic nanocarrier transitions to an active targeting state, exposing antigens and stimuli‐responsive ligands that facilitate macrophage recognition (ii), uptake (iii), and phagosomal trafficking (iv), thereby enabling localized intracellular drug release.

Researchers are now utilizing NPs coated with membranes derived from host cells, such as erythrocytes, leukocytes, or a combination thereof. These carriers exhibit “self” markers, such as CD47, which facilitate evasion from the immune system while targeting inflamed tissues or infections (Kelly et al. 2015; J. Li et al. 2023; Figure 5). Furthermore, the hybrid membranes present pathogen antigens, thereby eliciting macrophage uptake and initiating a targeted immune response (Chen et al. 2023). This methodology integrates immune evasion with immune activation, representing an advanced form of precision medicine that enables targeted drug delivery and immune stimulation.

#### 5.2.7 Biolistic Microdelivery: A Local Precision Platform

**Figure 6:**
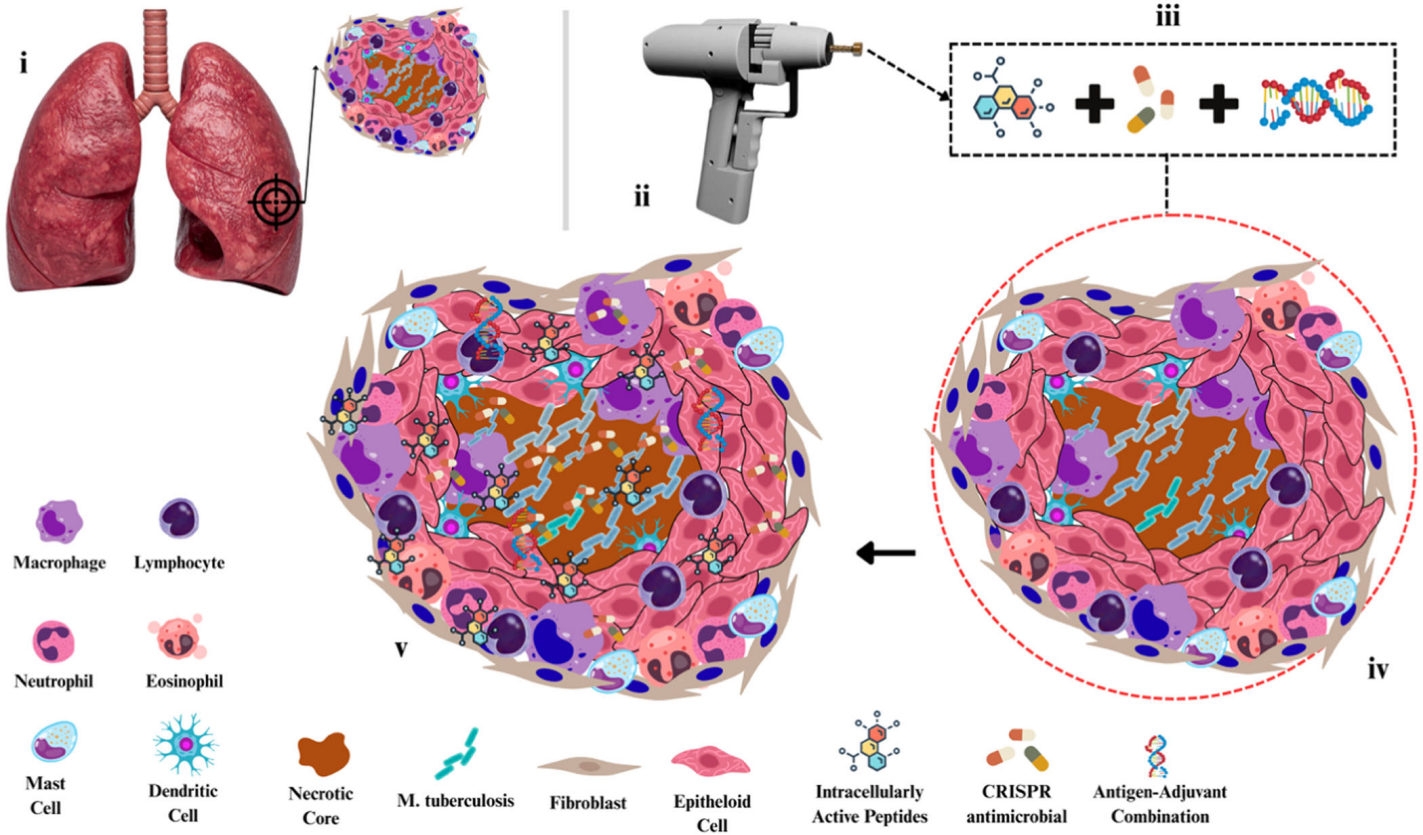
Biolistic microdelivery targeting granulomatous lesions in tuberculosis. (i) Localization of lung granuloma as the therapeutic target. (ii) Use of a biolistic device to deliver a combination pharmacological agent. (iii) Pharmacological agent composition: small‐molecule antibiotics, CRISPR antimicrobials, and antigen–adjuvant constructs. (iv) Untreated granuloma microenvironment with organized immune cell architecture. (v) Posttreatment state showing infiltrated therapeutic pharmacological agent designed to reprogram macrophages, promote autophagy, and eradicate *M. tuberculosis*.

Where systemic nanocarriers facilitate distribution, biolistic delivery systems offer localized precision and control (Tripathi et al. 2025; Simkhada et al. 2025; Figure 6). Modern gene gun platforms can accelerate microprojectiles coated with DNA, mRNA, adjuvants, or even CRISPR antimicrobials into tissue‐resident immune or epithelial cells (C. Zeballos and Gaj 2021). For persistent niches such as *M. tuberculosis* granulomas, *S. aureus* biofilm‐associated osteomyelitis, or mucosal *Chlamydia* infection, biolistic systems could locally deliver antigen–adjuvant combinations, pro‐autophagy activators, or intracellularly active peptides, acting as a spatiotemporal ignition switch for bacterial clearance (Kim et al. 2022; C. Wang et al. 2023; Xue et al. 2024). Coupling this with stimuli‐responsive coatings or synthetic gene circuits further restricts activity to inflamed microenvironments, aligning biolistic delivery with the broader precision‐medicine paradigm (Xue et al. 2024).

### 5.3 Artificial Intelligence and Systems Biology

Artificial Intelligence (AI) has emerged as a powerful tool capable of reshaping how we approach drug design and delivery for intracellular bacterial pathogens (Thomas et al. 2022). AI methods enable the rapid discovery of novel antimicrobial structures, the accurate prediction of drug–target interactions, and the precise optimization of PK/PD properties (Ocana et al. 2025). Deep learning (DL) models, generative algorithms, and reinforcement learning strategies can mine vast chemical and omics data sets to design molecules that address key challenges such as poor membrane penetration and efflux‐mediated clearance.

A compelling example is APEXDUO, an AI‐driven platform that utilizes DL‐based peptide design to generate antimicrobial peptides (AMPs) with an enhanced ability to penetrate host cells and kill intracellular bacteria (Cesaro et al. 2024; Abdel‐Rehim et al. 2025). Beyond drug design, AI is increasingly used to enhance drug delivery platforms. Machine learning (ML) models can simulate NP behavior, predict release kinetics under intracellular conditions, and optimize formulations to respond to pH or enzymatic triggers, thereby improving bioavailability and minimizing off‐target toxicity (Thomas et al. 2022).

#### 5.3.1 AI‐Driven Prediction Models

**Figure 7:**
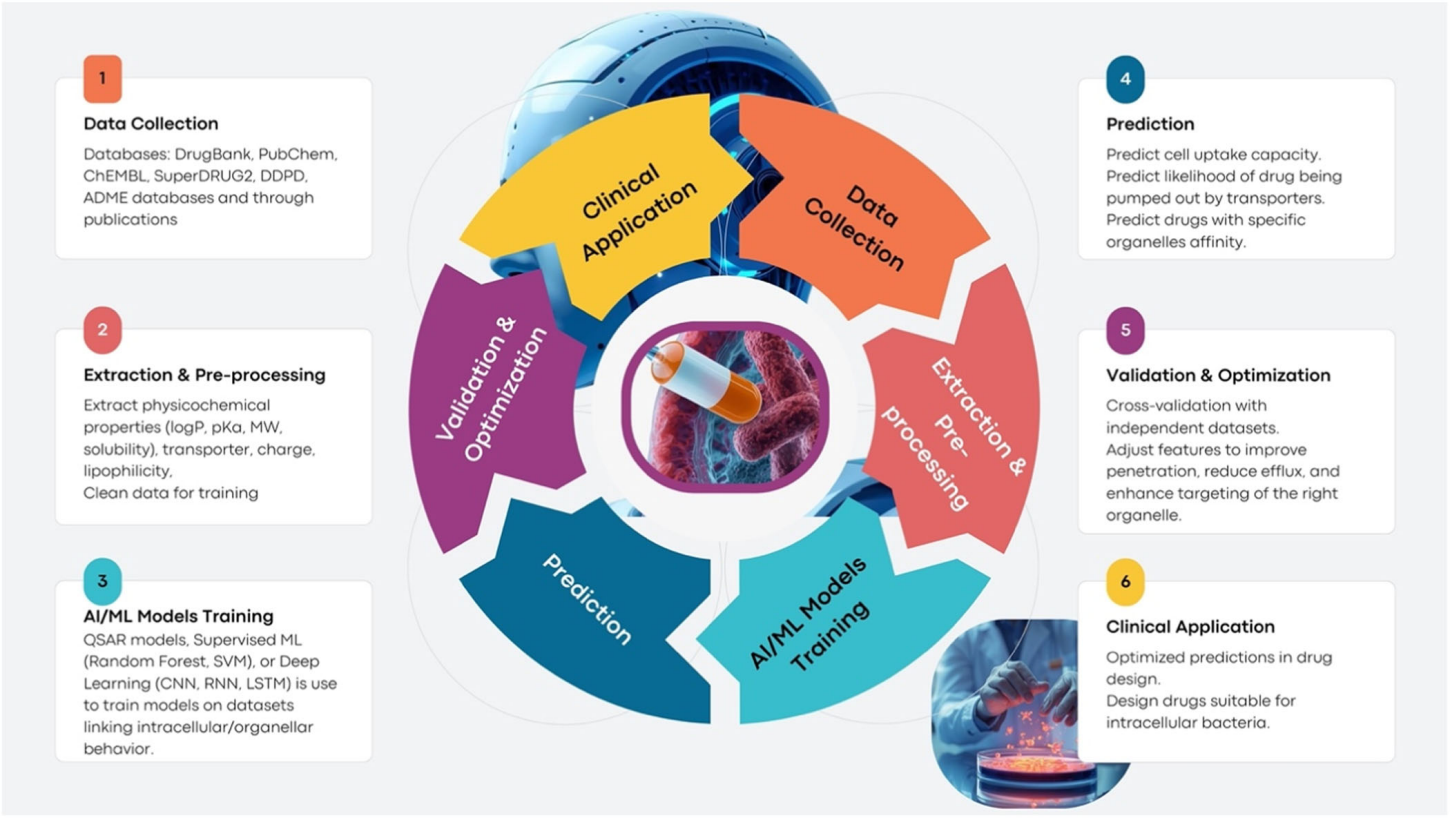
Stepwise schematics of an AI/ML‐guided framework for intracellular antibacterial drug design.

Future therapeutic development against intracellular bacterial pathogens will rely heavily on AI‐driven prediction models that account for barriers to cellular entry, efflux activity, and organelle‐specific targeting (Figure 7). Traditional drug design pipelines often fail to capture the complexity of intracellular PK. AI approaches—including random forest classifiers, support vector machines (SVM), neural networks, Bayesian inference models, and deep learning architectures (CNN, LSTM, RNN)—are now being deployed to predict PK parameters based on molecular descriptors and physicochemical properties (Hessler and Baringhaus 2018; Bhattamisra et al. 2023; Vora et al. 2023).

QSAR models remain central for ADMET prediction, including solubility, permeability, and metabolic stability (Daoui et al. 2021). However, next‐generation models must explicitly incorporate transporter‐mediated uptake and efflux processes, as these can dramatically alter intracellular drug disposition. Molecular properties such as lipophilicity, ionization (pKa), and susceptibility to efflux pumps like P‐glycoprotein are key determinants of effective intracellular penetration (Komura et al. 2023; Arav 2024). AI platforms such as APEXDUO exemplify this integration, having identified novel AMPs that significantly reduce intracellular *S. aureus* loads (Cesaro et al. 2024). In parallel, random forest classifiers and flexible molecular docking have been employed to predict efflux pump substrates (Ohashi et al. 2019), while regression‐based models (MLR, PLSR, SVM, RF, NN) are being developed to estimate organelle‐specific drug concentrations with high accuracy (X. Liu et al. 2024; Q. Wang et al. 2025).

#### 5.3.2 Machine Learning in NP Design

NPs are critical enablers of targeted delivery, improved intracellular bioavailability, and environmentally triggered release in acidic, redox‐rich, or enzyme‐active niches (Chen et al. 2023). Traditional NP design, however, is slow, labor‐intensive, and often relies on empirical trial‐and‐error approaches. Machine learning offers a data‐driven, iterative alternative that can accelerate NP design and optimization.

**Figure 8:**
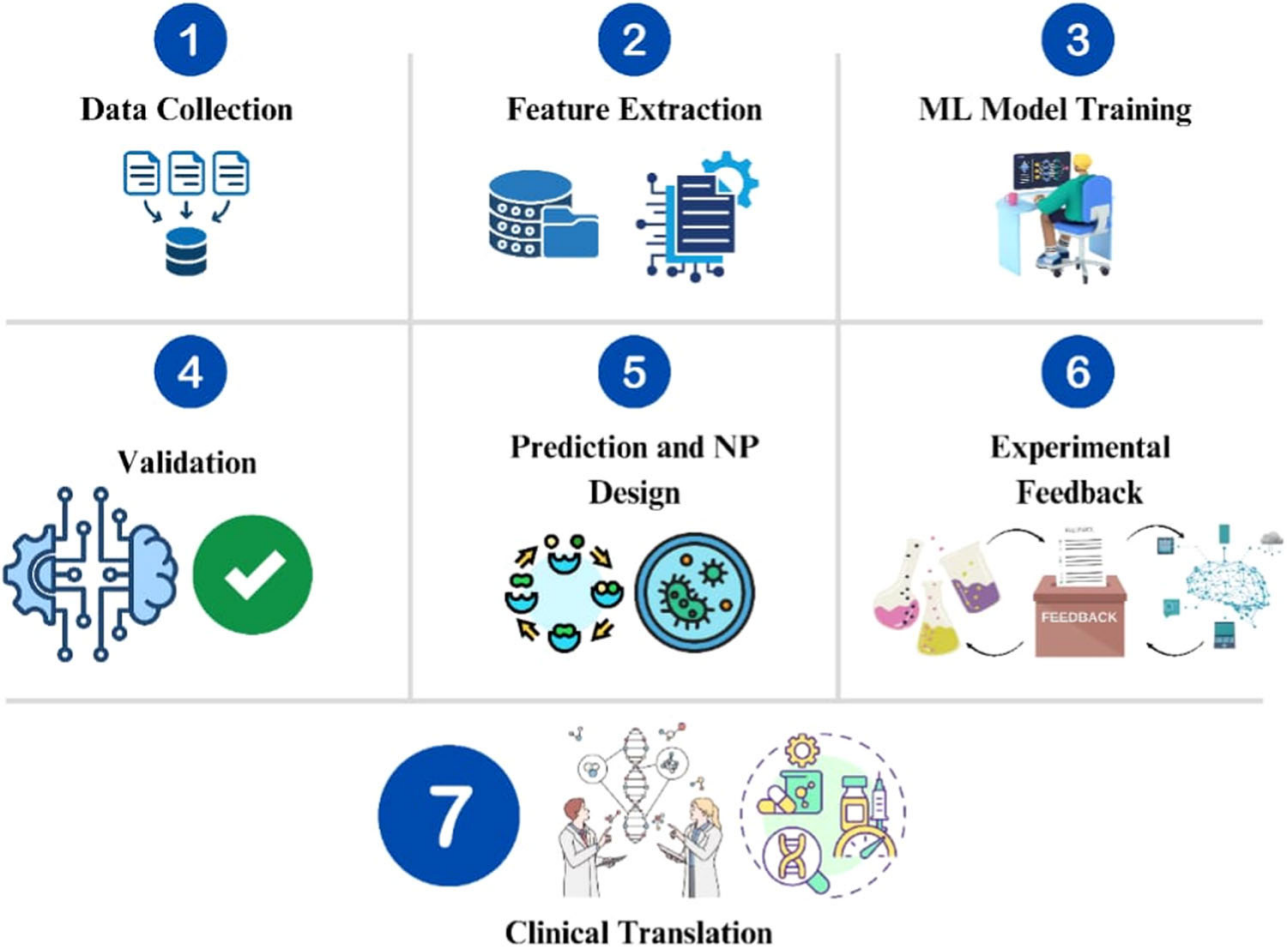
Stepwise schematics of a machine learning–guided pipeline for nanoparticle design and clinical translation.

Nanoinformatics databases such as NCL, NanoRegistry, NanoHUB, and NIKC provide curated physicochemical and biological data sets that fuel ML models. These models predict NP uptake, biodistribution, drug release profiles, and toxicity with increasing accuracy (Q. Wang et al. 2025). Regression and classification algorithms link synthesis parameters (e.g., polymer ratios, solvent conditions) to encapsulation efficiency and therapeutic performance, while deep learning approaches predict stimuli‐responsive release kinetics under pH or oxidative stress (Noorain et al. 2023). Optimized ML‐guided NPs have the potential to enhance phagocytic uptake, improve lysosomal escape, and deliver therapeutic agents to Intracellular bacterial pathogens with minimal collateral toxicity. This convergence of ML‐driven design and nanomedicine offers a blueprint for developing multifunctional nanocarriers that can reliably overcome intracellular delivery barriers and improve treatment outcomes for multidrug‐resistant infections. (Chen et al. 2023; Noorain et al. 2023; Q. Wang et al. 2025; Figure 8).

#### 5.3.3 Omics‐Guided Host‐Directed Therapies

Omics technologies have significantly advanced our comprehension of host–pathogen interactions by providing a systems‐level perspective across various omics fields, including genomics, transcriptomics, proteomics, metabolomics, and interactomics (Dai and Shen 2022). Technology‐driven omics methodologies, such as mass spectrometry and sequencing platforms, are employed to generate data, whereas knowledge‐driven omics—such as immunomics and microbiomics—detect immune epitopes and elucidate the contributions of the microbiome to disease pathogenesis (Subramanian et al. 2019; Babu and Snyder 2023).

Integrating multiple omics layers enables a deeper understanding of molecular complexity, revealing pathway interdependencies that genomics alone cannot reveal (Babu and Snyder 2023). Combining omics data with AI‐driven analytics is especially effective for developing host‐directed therapies (HDT). Multiomics data sets show host transcriptional reprogramming, proteomic interaction networks, and metabolic changes that support pathogen persistence. Machine learning algorithms can identify actionable signals from these data sets, turning high‐dimensional data into predictive models (Hasin et al. 2017; Misra et al. 2019; Ali 2023). For example, models that integrate SNP data and transcriptomics can predict host susceptibility to M. tuberculosis (Subramanian et al. 2020). Both unsupervised methods (such as matrix factorization and Bayesian network modeling) and supervised algorithms (like RF, SVM, and neural networks) are used to predict immune responses and stratify patients for HDT treatments (Winkle et al. 2021; Babu and Snyder 2023).

Artificial Intelligence‐driven prediction models, machine learning‐guided nanocarrier design, and omics‐informed host‐directed strategies establish a synergistic framework for the development of next‐generation therapies targeting intracellular bacterial pathogens (Dai and Shen 2022; Thomas et al. 2022; Babu and Snyder 2023; Ocana et al. 2025). These tools foster a rational design ecosystem that correlates molecular descriptors with intracellular PK/PD predictions, enhances carrier systems for precise targeted delivery, and integrates host biological factors into therapeutic decision‐making processes (Komura et al. 2023; Noorain et al. 2023; Q. Wang et al. 2025). This comprehensive strategy facilitates not only the creation of drugs capable of superior intracellular penetration but also their accurate spatiotemporal deployment and alignment with immunological responses, thereby advancing durable bacterial eradication, preventing relapse, and mitigating resistance (Mishra et al. 2011; N. Tian, Chu et al. 2025; Tao et al. 2025)

## 6 Conclusion

Complementary strategies, including stimuli‐responsive nanocarriers (Rao et al. 2022; J. Li et al. 2023), outer membrane vesicle (OMV)‐coated systems (Colilla and Vallet‐Regí 2020; Fazal and Lee 2021), biomimetic cloaks such as red blood cell– and leukocyte‐derived carriers (L. Zhang et al. 2022; Chen et al. 2023), dual‐agent host‐directed therapy platforms that co‐deliver antibiotics and immune modulators (Xue et al. 2024; N. Tian, Chu et al. 2025), and programmable biolistic or gene‐gun delivery systems engineered for spatiotemporal precision (V. Kumar et al. 2024), are converging into a cohesive, systems‐level therapeutic framework for tackling intracellular bacterial infections.

The future of managing persistent intracellular bacterial pathogens will not rely on a singular “magic bullet.” Instead, it should be based on precisely coordinated, multimodal regimens capable of dynamically sensing infection microenvironments, homing to specific subcellular niches, and adaptively responding to pathogen‐driven immune modulation (Alikhani et al. 2025). By integrating targeted drug delivery with immune reprogramming, phagosomal pH and redox‐responsive release mechanisms, and microenvironmental engineering, these strategies promise not only rapid pathogen eradication but also enduring immune recalibration, relapse prevention, and mitigation of antimicrobial resistance (Pilla‐Moffett et al. 2016; Kaufmann et al. 2018).

Collectively, the strategies highlighted in this review signal a paradigm shift away from traditional antimicrobial administration and toward a precision medicine–guided, spatiotemporally intelligent therapeutic model, one that actively engages the host–pathogen interface and redefines the treatment of persistent intracellular bacterial infections.

## Author Contributions

**Babatunde Ibrahim Olowu:** conceptualization, writing – original draft, figure preparation, writing – review and editing, writing – final manuscript. **Maryam Ebunoluwa Zakariya:** writing – original draft. **Abdulmuheez Abiola Abdulkareem:** writing – original draft. **Olalekan Toheeb Okewale:** writing – original draft, figure preparation and visualization. **Muhammad Halima Idris:** figure preparation and visualization. **Halimah Oluwayemisi Olayiwola:** writing – original draft.

## Conflicts of Interest

The authors declare no conflicts of interest.

## Data Availability

Data sharing not applicable to this article as no data sets were generated or analyzed during the current study.

## References

[ddr70198-bib-0001] Abdel‐Rehim, A. , O. Orhobor , G. Griffiths , L. Soldatova , and R. D. King . 2025. “Establishing Predictive Machine Learning Models for Drug Responses in Patient Derived Cell Culture.” Npj Precision Oncology 9, no. 1: 180. 10.1038/S41698-025-00937-2;SUBJMETA. 40514399 PMC12166088

[ddr70198-bib-0002] Abel Zur Wiesch, P. , F. Clarelli , and T. Cohen . 2017. “Using Chemical Reaction Kinetics to Predict Optimal Antibiotic Treatment Strategies.” PLoS Computational Biology 13, no. 1: 1005321. 10.1371/journal.pcbi.1005321. PMC5257006 28060813

[ddr70198-bib-0003] Ali, H. 2023. “Artificial Intelligence in Multi‐Omics Data Integration: Advancing Precision Medicine, Biomarker Discovery, and Genomic‐Driven Disease Interventions.” ResearchGate. https://www.researchgate.net/publication/389556142.

[ddr70198-bib-0004] Alikhani, M. S. , M. Nazari , and S. Hatamkhani . 2025. “Enhancing Antibiotic Therapy Through Comprehensive Pharmacokinetic/Pharmacodynamic Principles.” Frontiers in Cellular and Infection Microbiology 15: 1521091. 10.3389/fcimb.2025.1521091. 40070375 PMC11893874

[ddr70198-bib-0005] Aljayyoussi, G. , V. A. Jenkins , R. Sharma , et al. 2017. “Pharmacokinetic‐Pharmacodynamic Modelling of Intracellular *Mycobacterium tuberculosis* Growth and Kill Rates Is Predictive of Clinical Treatment Duration.” Scientific Reports 7: 502. 10.1038/s41598-017-00529-6. 28356552 PMC5428680

[ddr70198-bib-0006] Allwood, E. M. , R. J. Devenish , M. Prescott , B. Adler , and J. D. Boyce . 2011. “Strategies for Intracellular Survival of Burkholderia Pseudomallei.” Frontiers in Microbiology 2: 170. 10.3389/fmicb.2011.00170. 22007185 PMC3159172

[ddr70198-bib-0007] Arav, Y. 2024. “Advances in Modeling Approaches for Oral Drug Delivery: Artificial Intelligence, Physiologically‐Based Pharmacokinetics, and First‐Principles Models.” Pharmaceutics 16, no. 8: 978. 10.3390/PHARMACEUTICS16080978. 39204323 PMC11359797

[ddr70198-bib-0008] Armenia, I. , C. Cuestas Ayllón , B. Torres Herrero , et al. 2022. “Photonic and Magnetic Materials for On‐Demand Local Drug Delivery.” Advanced Drug Delivery Reviews 191: 114584. 10.1016/j.addr.2022.114584. 36273514

[ddr70198-bib-0009] Arnett, E. , R. I. Lehrer , P. Pratikhya , W. Lu , and S. Seveau . 2011. “Defensins Enable Macrophages to Inhibit the Intracellular Proliferation of *Listeria monocytogenes* .” Cellular Microbiology 13, no. 4: 635–651. 10.1111/J.1462-5822.2010.01563.X. 21143570

[ddr70198-bib-0010] Aulicino, A. , A. Antanaviciute , J. Frost , et al. 2022. “Dual RNA Sequencing Reveals Dendritic Cell Reprogramming in Response to Typhoidal Salmonella Invasion.” Communications Biology 5: 111. 10.1038/s42003-022-03038-z. 35121793 PMC8816929

[ddr70198-bib-0011] Aulner, N. , A. Danckaert , J. Ihm , D. Shum , and S. L. Shorte . 2019. “Next‐Generation Phenotypic Screening in Early Drug Discovery for Infectious Diseases.” Trends in Parasitology 35, no. 7: 559–570. 10.1016/j.pt.2019.05.004. 31176583

[ddr70198-bib-0012] Avanzi, C. , P. Singh , R. W. Truman , and P. N. Suffys . 2020. “Molecular Epidemiology of Leprosy: An Update.” Infection, Genetics and Evolution 86: 104581. 10.1016/j.meegid.2020.104581. 33022427

[ddr70198-bib-0013] Avital, G. , R. Avraham , A. Fan , T. Hashimshony , D. T. Hung , and I. Yanai . 2017. “scDual‐Seq: Mapping the Gene Regulatory Program of Salmonella Infection by Host and Pathogen Single‐Cell RNA‐Sequencing.” Genome Biology 18: 200. 10.1186/s13059-017-1340-x. 29073931 PMC5658913

[ddr70198-bib-0014] Babu, M. , and M. Snyder . 2023. “Multi‐Omics Profiling for Health.” Molecular & Cellular Proteomics: MCP 22, no. 6: 100561. 37119971 10.1016/j.mcpro.2023.100561 PMC10220275

[ddr70198-bib-0015] Batalha, I. L. , A. Bernut , M. Schiebler , et al. 2019. “Polymeric Nanobiotics as a Novel Treatment for Mycobacterial Infections.” Journal of Controlled Release 314: 116–124. 10.1016/J.JCONREL.2019.10.009. 31647980 PMC6899522

[ddr70198-bib-0016] Bhattamisra, S. K. , P. Banerjee , P. Gupta , J. Mayuren , S. Patra , and M. Candasamy . 2023. “Artificial Intelligence in Pharmaceutical and Healthcare Research.” Big Data and Cognitive Computing 7, no. 1: 10. 10.3390/BDCC7010010.

[ddr70198-bib-0017] Buccini, D. F. , M. H. Cardoso , and O. L. Franco . 2021. “Antimicrobial Peptides and Cell‐Penetrating Peptides for Treating Intracellular Bacterial Infections.” Frontiers in Cellular and Infection Microbiology 10: 612931. 10.3389/FCIMB.2020.612931. 33614528 PMC7892433

[ddr70198-bib-0018] Bueno, S. M. , P. A. González , L. J. Carreño , et al. 2008. “The Capacity Ofsalmonellato Survive Inside Dendritic Cells and Prevent Antigen Presentation to T Cells Is Host Specific.” Immunology 124, no. 4: 522–533. 10.1111/j.1365-2567.2008.02805.x. 18266715 PMC2492944

[ddr70198-bib-0019] Cai, Y. , M. Qiao , X. Xie , et al. 2025. “Intracellular MRSA‐Targeted Lipid‐Polymer Hybrid Nanoparticles for Macrophage Reprogramming and Intracellular MRSA Eradication.” Chemical Engineering Journal 505: 159665. 10.1016/J.CEJ.2025.159665.

[ddr70198-bib-0020] Carryn, S. , F. Van Bambeke , M. P. Mingeot‐Leclercq , and P. M. Tulkens . 2002. “Comparative Intracellular (THP‐1 Macrophage) and Extracellular Activities of β‐Lactams, Azithromycin, Gentamicin, and Fluoroquinolones Against *Listeria monocytogenes* at Clinically Relevant Concentrations.” Antimicrobial Agents and Chemotherapy 46, no. 7: 2095–2103. 10.1128/AAC.46.7.2095-2103.2002. 12069960 PMC127291

[ddr70198-bib-0021] Caven, L. T. , A. J. Brinkworth , and R. A. Carabeo . 2023. “Chlamydia Trachomatis Induces the Transcriptional Activity of Host YAP in a Hippo‐Independent Fashion.” Frontiers in Cellular and Infection Microbiology 13: 1098420. 10.3389/FCIMB.2023.1098420. 36923592 PMC10008951

[ddr70198-bib-0022] Celli, J. 2019. “The Intracellular Life Cycle of Brucella spp.” Microbiology Spectrum 7, no. 10: 1128. 10.1128/microbiolspec.bai-0006-2019. PMC6448592 30848234

[ddr70198-bib-0023] Cesaro, A. , F. Wan , M. D. T. Torres , and C. de la Fuente‐Nunez . 2024. “Design of Multimodal Antibiotics Against Intracellular Infections Using Deep Learning.” BioRxiv 2024.12.20.629780. 10.1101/2024.12.20.629780.

[ddr70198-bib-0024] Chan, A. , and A. Tsourkas . 2024. “Intracellular Protein Delivery: Approaches, Challenges, and Clinical Applications.” BME Frontiers 5: 0035. 10.34133/bmef.0035. 38282957 PMC10809898

[ddr70198-bib-0025] Chandra, P. , S. J. Grigsby , and J. A. Philips . 2022. “Immune Evasion and Provocation by *Mycobacterium tuberculosis* .” Nature Reviews Microbiology 20, no. 12: 750–766. 10.1038/s41579-022-00763-4. 35879556 PMC9310001

[ddr70198-bib-0026] Chen, S.‐Y. , X.‐X. Xu , X. Li , et al. 2022. “Recent Advances in the Intracellular Delivery of Macromolecule Therapeutics.” Biomaterials Science 10: 6642–6655. 10.1039/D2BM01348G. 36214257

[ddr70198-bib-0027] Chen, Y. , X. He , Q. Chen , et al. 2023. “Nanomaterials Against Intracellular Bacterial Infection: From Drug Delivery to Intrinsic Biofunction.” Frontiers in Bioengineering and Biotechnology 11: 1197974. 10.3389/fbioe.2023.1197974. 37180049 PMC10174311

[ddr70198-bib-0028] Chifiriuc, M. C. , A. M. Holban , C. Curutiu , et al. 2016. “Antibiotic Drug Delivery Systems for the Intracellular Targeting of Bacterial Pathogens.” Smart Drug Delivery System. 10.5772/61327.

[ddr70198-bib-0029] Chu, S. , X. Shi , Y. Tian , and F. Gao . 2022. “pH‐Responsive Polymer Nanomaterials for Tumor Therapy.” Frontiers in Oncology 12: 855019. 10.3389/fonc.2022.855019. 35392227 PMC8980858

[ddr70198-bib-0030] Clemente, T. M. , R. K. Angara , and S. D. Gilk . 2023. “Establishing the Intracellular Niche of Obligate Intracellular Vacuolar Pathogens.” Frontiers in Cellular and Infection Microbiology 13: 1206037. 10.3389/fcimb.2023.1206037. 37645379 PMC10461009

[ddr70198-bib-0031] Colilla, M. , and M. Vallet‐Regí . 2020. “Targeted Stimuli‐Responsive Mesoporous Silica Nanoparticles for Bacterial Infection Treatment.” International Journal of Molecular Sciences 21, no. 22: 8605. 10.3390/ijms21228605. 33203098 PMC7696808

[ddr70198-bib-0032] Colonne, P. M. , C. G. Winchell , and D. E. Voth . 2016. “Hijacking Host Cell Highways: Manipulation of the Host Actin Cytoskeleton by Obligate Intracellular Bacterial Pathogens.” Frontiers in Cellular and Infection Microbiology 6: 107. 10.3389/fcimb.2016.00107. 27713866 PMC5031698

[ddr70198-bib-0033] Compagne, N. , A. Vieira Da Cruz , R. T. Müller , R. C. Hartkoorn , M. Flipo , and K. M. Pos . 2023. “Update on the Discovery of Efflux Pump Inhibitors Against Critical Priority Gram‐Negative Bacteria.” Antibiotics (USSR) 12, no. 1: 180. 10.3390/antibiotics12010180. PMC9854755 36671381

[ddr70198-bib-0034] Cresti, L. , G. Cappello , and A. Pini . 2024. “Antimicrobial Peptides Towards Clinical Application—A Long History to Be Concluded.” International Journal of Molecular Sciences 25, no. 9: 4870. 10.3390/IJMS25094870. 38732089 PMC11084544

[ddr70198-bib-0035] Croston, G. E. 2017. “The Utility of Target‐Based Discovery.” Expert Opinion on Drug Discovery 12, no. 5: 427–429. 10.1080/17460441.2017.1308351. 28306350

[ddr70198-bib-0036] Dai, X. , and L. Shen . 2022. “Advances and Trends in Omics Technology Development.” Frontiers in Medicine 9: 911861. 35860739 10.3389/fmed.2022.911861 PMC9289742

[ddr70198-bib-0037] Daoui, O. , S. Elkhattabi , S. Chtita , R. Elkhalabi , H. Zgou , and A. T. Benjelloun . 2021. “QSAR, Molecular Docking and ADMET Properties In Silico Studies of Novel 4,5,6,7‐tetrahydrobenzo[D]‐thiazol‐2‐Yl Derivatives Derived From Dimedone as Potent Anti‐Tumor Agents Through Inhibition of C‐Met Receptor Tyrosine Kinase.” Heliyon 7, no. 7: e07463. 10.1016/J.HELIYON.2021.E07463. 34296007 PMC8282965

[ddr70198-bib-0038] Dassonville‐Klimpt, A. , and P. Sonnet . 2020. “Advances in ‘Trojan Horse' Strategies in Antibiotic Delivery Systems.” Future Medicinal Chemistry 12, no. 11: 983–986. 10.4155/fmc-2020-0065. 32270702

[ddr70198-bib-0039] Day, N. J. , P. Santucci , and M. G. Gutierrez . 2024. “Host Cell Environments and Antibiotic Efficacy in Tuberculosis.” Trends in Microbiology 32, no. 3: 270–279. 10.1016/j.tim.2023.08.009. 37709598

[ddr70198-bib-0040] De Duve, C. , T. De Barsy , B. Poole , A. Trouet , P. Tulkens , and F. Van Hoof . 1974. “Lysosomotropic Agents.” Biochemical Pharmacology 23, no. 18: 2495–2531. 10.1016/0006-2952(74)90174-9. 4606365

[ddr70198-bib-0041] de Figueiredo, P. , T. A. Ficht , A. Rice‐Ficht , C. A. Rossetti , and L. G. Adams . 2015. “Pathogenesis and Immunobiology of Brucellosis.” American Journal of Pathology 185, no. 6: 1505–1517. 10.1016/j.ajpath.2015.03.003. 25892682 PMC4450313

[ddr70198-bib-0042] Dereje, D. M. , F. Bianco , C. Pontremoli , A. Fiorio Pla , and N. Barbero . 2025. “NIR pH‐Responsive PEGelated Plga Nanoparticles as Effective Phototoxic Agents in Resistant PDAC Cells.” Polymers 17, no. 8: 1101. 10.3390/polym17081101. 40284366 PMC12030558

[ddr70198-bib-0043] Derendorf, H. 2020. “Excessive Lysosomal Ion‐Trapping of Hydroxychloroquine and Azithromycin.” International Journal of Antimicrobial Agents 55, no. 6: 106007. 10.1016/j.ijantimicag.2020.106007. 32389720 PMC7204663

[ddr70198-bib-0044] Desai, N. , D. Rana , S. Salave , D. Benival , D. Khunt , and B. G. Prajapati . 2024. “Achieving Endo/Lysosomal Escape Using Smart Nanosystems for Efficient Cellular Delivery.” Molecules 29, no. 13: 3131. 10.3390/molecules29133131. 38999083 PMC11243486

[ddr70198-bib-0045] Donnellan, S. , G. Aljayyoussi , E. Moyo , et al. 2019. “Intracellular Pharmacodynamic Modeling Is Predictive of the Clinical Activity of Fluoroquinolones Against Tuberculosis.” Antimicrobial Agents and Chemotherapy 64, no. 1: 919–989. 10.1128/AAC.00989-19. PMC7187570 31611354

[ddr70198-bib-0046] Eke, I. E. , and R. B. Abramovitch . 2025. “Functions of Nitroreductases in Mycobacterial Physiology and Drug Susceptibility.” Journal of Bacteriology 207, no. 2: 324–326. 10.1128/jb.00326-24. PMC11841060 39772630

[ddr70198-bib-0047] Eldridge, M. J. G. , and M. A. Hamon . 2021. “Histone H3 Deacetylation Promotes Host Cell Viability for Efficient Infection by *Listeria monocytogenes* .” PLoS Pathogens 17, no. 12: e1010173. 10.1371/JOURNAL.PPAT.1010173. 34929015 PMC8722725

[ddr70198-bib-0048] Ellis, M. J. , C. N. Tsai , J. W. Johnson , et al. 2019. “A Macrophage‐Based Screen Identifies Antibacterial Compounds Selective for Intracellular Salmonella Typhimurium.” Nature Communications 10, no. 1: 197. 10.1038/s41467-018-08190-x. PMC6331611 30643129

[ddr70198-bib-0049] Elsharkasy, O. M. , J. Z. Nordin , D. W. Hagey , et al. 2020. “Extracellular Vesicles as Drug Delivery Systems: Why and How?.” Advanced Drug Delivery Reviews 159: 332–343. 10.1016/j.addr.2020.04.004. 32305351

[ddr70198-bib-0050] Elwell, C. , K. Mirrashidi , and J. Engel . 2016. “Chlamydia Cell Biology and Pathogenesis.” Nature Reviews Microbiology 14, no. 6: 385–400. 10.1038/nrmicro.2016.30. 27108705 PMC4886739

[ddr70198-bib-0051] Ertl, P. 2007. “Polar Surface Area.” In Methods and Principles in Medicinal Chemistry (1st ed.), edited by In. R. Mannhold , 111–126. Wiley. 10.1002/9783527621286.ch5.

[ddr70198-bib-0052] Escoll, P. , O. R. Song , F. Viana , et al. 2017. “Legionella Pneumophila Modulates Mitochondrial Dynamics.” Cell Host & Microbe 22, no. 3: 302–316.e7. 7. 10.1016/j.chom.2017.07.020. 28867389

[ddr70198-bib-0053] Eskeland, S. , E. G. Bø‐Granquist , S. Stuen , et al. 2023. “Temporal Patterns of Gene Expression in Response to Inoculation With a Virulent Anaplasma phagocytophilum Strain in Sheep.” Scientific Reports 13: 20399. 10.1038/s41598-023-47801-6. 37989861 PMC10663591

[ddr70198-bib-0054] Ezeh, C. K. , and M. Dibua . 2024. “Anti‐Biofilm, Drug Delivery and Cytotoxicity Properties of Dendrimers.” ADMET & DMPK 12, no. 2: 239–267. 10.5599/ADMET.1917. 38720923 PMC11075165

[ddr70198-bib-0055] Fan, J. , and I. A. M. de Lannoy . 2014. “Pharmacokinetics.” Biochemical Pharmacology 87, no. 1: 93–120. 10.1016/j.bcp.2013.09.007. 24055064

[ddr70198-bib-0056] Fang, L. , H. Zhou , L. Cheng , Y. Wang , F. Liu , and S. Wang . 2023. “The Application of Mesoporous Silica Nanoparticles as a Drug Delivery Vehicle in Oral Disease Treatment.” Frontiers in Cellular and Infection Microbiology 13: 1124411. 10.3389/fcimb.2023.1124411. 36864881 PMC9971568

[ddr70198-bib-0057] Fazal, S. , and R. Lee . 2021. “Biomimetic Bacterial Membrane Vesicles for Drug Delivery Applications.” Pharmaceutics 13, no. 9: 1430. 10.3390/pharmaceutics13091430. 34575506 PMC8468068

[ddr70198-bib-0058] Fortuni, B. , T. Inose , M. Ricci , et al. 2019. “Polymeric Engineering of Nanoparticles for Highly Efficient Multifunctional Drug Delivery Systems.” Scientific Reports 9, no. 1: 2666. 10.1038/s41598-019-39107-3. 30804375 PMC6389875

[ddr70198-bib-0059] Gaurav, A. , P. Bakht , M. Saini , S. Pandey , and R. Pathania . 2023. “Role of Bacterial Efflux Pumps in Antibiotic Resistance, Virulence, and Strategies to Discover Novel Efflux Pump Inhibitors.” Microbiology 169, no. 5: 001333. 10.1099/mic.0.001333. 37224055 PMC10268834

[ddr70198-bib-0060] Ghosh, R. , and M. De . 2023. Liposome‐Based Antibacterial Delivery: An Emergent Approach to Combat Bacterial Infections. 10.1021/acsomega.3c04893. PMC10551917 37810644

[ddr70198-bib-0061] Ghosh, S. , and T. J. O'Connor . 2017. “Beyond Paralogs: The Multiple Layers of Redundancy in Bacterial Pathogenesis.” Frontiers in Cellular and Infection Microbiology 7: 467. 10.3389/fcimb.2017.00467. 29188194 PMC5694747

[ddr70198-bib-0062] Gomarasca, M. , T. F. C. Martins , L. Greune , P. R. Hardwidge , M. A. Schmidt , and C. Rüter . 2017. “Bacterium‐Derived Cell‐Penetrating Peptides Deliver Gentamicin to Kill Intracellular Pathogens.” Antimicrobial Agents and Chemotherapy 61, no. 4: e02545‐16. 10.1128/AAC.02545-16. 28096156 PMC5365713

[ddr70198-bib-0063] Górska, A. , A. Sloderbach , and M. P. Marszałł . 2014. “Siderophore–Drug Complexes: Potential Medicinal Applications of the ‘Trojan Horse' Strategy.” Trends in Pharmacological Sciences 35, no. 9: 442–449. 10.1016/j.tips.2014.06.007. 25108321

[ddr70198-bib-0064] Gräff, Á. T. , and S. M. Barry . 2024. “Siderophores as Tools and Treatments.” NPJ Antimicrobials and Resistance 2, no. 1: 47. 10.1038/s44259-024-00053-4. 39649077 PMC11621027

[ddr70198-bib-0065] Guidi‐Rontani, C. , M. Levy , H. Ohayon , and M. Mock . 2001. “Fate of Germinated Bacillus Anthracis Spores in Primary Murine Macrophages.” Molecular Microbiology 42, no. 4: 931–938. 10.1046/j.1365-2958.2001.02695.x. 11737637

[ddr70198-bib-0066] Gunn, N. J. , S. P. Kidd , L. B. Solomon , D. Yang , E. Roscioli , and G. J. Atkins . 2024. “ *Staphylococcus aureus* Persistence in Osteocytes: Weathering the Storm of Antibiotics and Autophagy/Xenophagy.” Frontiers in Cellular and Infection Microbiology 14: 1403289. 10.3389/fcimb.2024.1403289. 38915921 PMC11194354

[ddr70198-bib-0067] Guo, X. , Y. Cheng , X. Zhao , Y. Luo , J. Chen , and W. E. Yuan . 2018. “Advances In Redox‐Responsive Drug Delivery Systems.” Journal of Nanobiotechnology 16: 74. 10.1186/s12951-018-0398-2. 30243297 PMC6151045

[ddr70198-bib-0068] Hanoulle, X. , J.‐M. Wieruszeski , P. Rousselot‐Pailley , et al. 2006. “Selective Intracellular Accumulation of the Major Metabolite Issued From the Activation of the Prodrug Ethionamide in Mycobacteria.” Journal of Antimicrobial Chemotherapy 58, no. 4: 768–772. 10.1093/jac/dkl332. 16895935

[ddr70198-bib-0069] Harms, A. , and C. Dehio . 2012. “Intruders below the Radar: Molecular Pathogenesis of Bartonella spp.” Clinical Microbiology Reviews 25, no. 1: 42–78. 10.1128/CMR.05009-11. 22232371 PMC3255967

[ddr70198-bib-0070] Hasin, Y. , M. Seldin , and A. Lusis . 2017. “Multi‐Omics Approaches to Disease.” Genome Biology 18, no. 1: 83. 10.1186/s13059-017-1215-1. 28476144 PMC5418815

[ddr70198-bib-0071] He, H. , Y. Lu , J. Qi , Q. Zhu , Z. Chen , and W. Wu . 2019. “Adapting Liposomes for Oral Drug Delivery.” Acta Pharmaceutica Sinica B 9, no. 1: 36–48. 10.1016/J.APSB.2018.06.005. 30766776 PMC6362257

[ddr70198-bib-0372] Herb, M. , V. Schatz , K. Hadrian , et al. 2024. “Macrophage Variants in Laboratory Research: Most Are Well Done, but Some Are RAW.” Frontiers in Cellular and Infection Microbiology 14: 1457323. 10.3389/fcimb.2024.1457323. 39445217 PMC11496307

[ddr70198-bib-0072] Herbst, C. , L. A. Harshyne , and B. Z. Igyártó . 2022. “Intracellular Monitoring by Dendritic Cells—A New Way to Stay Informed.” Frontiers in Immunology 13: 1053582. 10.3389/fimmu.2022.1053582. 36389660 PMC9647004

[ddr70198-bib-0073] Hessler, G. , and K. H. Baringhaus . 2018. “Artificial Intelligence in Drug Design.” Molecules 23, no. 10: 2520. 10.3390/MOLECULES23102520. 30279331 PMC6222615

[ddr70198-bib-0074] Hosseini, S. M. , M. Taheri , F. Nouri , A. Farmani , N. M. Moez , and M. R. Arabestani . 2022. “Nano Drug Delivery in Intracellular Bacterial Infection Treatments.” Biomedicine & Pharmacotherapy = Biomedecine & Pharmacotherapie 146: 112609. 10.1016/J.BIOPHA.2021.112609. 35062073

[ddr70198-bib-0075] Huang, D. , J. Luo , X. OuYang , and L. Song . 2022. “Subversion of Host Cell Signaling: The Arsenal of Rickettsial Species.” Frontiers in Cellular and Infection Microbiology 12: 995933. 10.3389/fcimb.2022.995933. 36389139 PMC9659576

[ddr70198-bib-0076] Huttunen, K. M. , H. Raunio , and J. Rautio . 2011. “Prodrugs—From Serendipity to Rational Design.” Pharmacological Reviews 63, no. 3: 750–771. 10.1124/pr.110.003459. 21737530

[ddr70198-bib-0077] Jensen, K. , I. J. Gallagher , A. Kaliszewska , et al. 2016. “Live and Inactivated *Salmonella enterica* Serovar Typhimurium Stimulate Similar but Distinct Transcriptome Profiles in Bovine Macrophages and Dendritic Cells.” Veterinary Research 47: 46. 10.1186/s13567-016-0328-y. 27000047 PMC4802613

[ddr70198-bib-0078] Jiang, L. , H. W. Lee , and S. C. J. Loo . 2020. “Therapeutic Lipid‐Coated Hybrid Nanoparticles Against Bacterial Infections.” RSC Advances 10, no. 14: 8497–8517. 10.1039/C9RA10921H. 35497832 PMC9050015

[ddr70198-bib-0079] Jubeh, B. , Z. Breijyeh , and R. Karaman . 2020. “Antibacterial Prodrugs to Overcome Bacterial Resistance.” Molecules 25, no. 7: 1543. 10.3390/molecules25071543. 32231026 PMC7180472

[ddr70198-bib-0080] Junyaprasert, V. B. , and P. Thummarati . 2023. “Innovative Design of Targeted Nanoparticles: Polymer‐Drug Conjugates for Enhanced Cancer Therapy.” Pharmaceutics 15, no. 9: 2216. 10.3390/pharmaceutics15092216. 37765185 PMC10537251

[ddr70198-bib-0081] Kamaruzzaman, N. F. , S. Kendall , and L. Good . 2017. “Targeting the Hard to Reach: Challenges and Novel Strategies in the Treatment of Intracellular Bacterial Infections.” British Journal of Pharmacology 174, no. 14: 2225–2236. 10.1111/bph.13664. 27925153 PMC5481648

[ddr70198-bib-0082] Karsdal, M. , T. R. Cox , A. L. Parker , et al. 2025. “Advances in Extracellular Matrix‐Associated Diagnostics and Therapeutics.” Journal of Clinical Medicine 14, no. 6: 1856. 10.3390/jcm14061856. 40142664 PMC11943371

[ddr70198-bib-0083] Kaufmann, S. H. E. , A. Dorhoi , R. S. Hotchkiss , and R. Bartenschlager . 2018. “Host‐Directed Therapies for Bacterial and Viral Infections.” Nature Reviews Drug Discovery 17, no. 1: 35–56. 10.1038/nrd.2017.162. 28935918 PMC7097079

[ddr70198-bib-0084] Kellermann, M. , F. Scharte , and M. Hensel . 2021. “Manipulation of Host Cell Organelles by Intracellular Pathogens.” International Journal of Molecular Sciences 22, no. 12: 6484. 10.3390/ijms22126484. 34204285 PMC8235465

[ddr70198-bib-0085] Kelly, P. M. , C. Åberg , E. Polo , et al. 2015. “Mapping Protein Binding Sites on the Biomolecular Corona of Nanoparticles.” Nature Nanotechnology 10: 472–479. 10.1038/nnano.2015.47. 25822932

[ddr70198-bib-0086] Khare, G. , P. Kumar , and A. K. Tyagi . 2013. “Whole‐Cell Screening‐Based Identification of Inhibitors Against the Intraphagosomal Survival of *Mycobacterium tuberculosis* .” Antimicrobial Agents and Chemotherapy 57, no. 12: 6372–6377. 10.1128/AAC.01444-13. 24060878 PMC3837871

[ddr70198-bib-0087] Khoei, A. , R. Soltani , J. Emami , S. Badri , and S. Taheri . 2019. “Therapeutic Drug Monitoring of Vancomycin by AUCτ‐MIC Ratio in Patients With Chronic Kidney Disease.” Research in Pharmaceutical Sciences 14, no. 1: 84–92. 10.4103/1735-5362.251856. 30936936 PMC6407338

[ddr70198-bib-0088] Kim, S.‐I. , J. Y. Ha , S.‐Y. Choi , S.‐H. Hong , and H.‐J. Lee . 2022. “Use of Bacterial Extracellular Vesicles for Gene Delivery to Host Cells.” Biomolecules 12, no. 9: 1171. 10.3390/BIOM12091171. 36139009 PMC9496234

[ddr70198-bib-0089] Klipp, A. , M. Burger , and J. C. Leroux . 2023. “Get out or Die Trying: Peptide‐ and Protein‐Based Endosomal Escape of RNA Therapeutics.” Advanced Drug Delivery Reviews 200: 115047. 10.1016/J.ADDR.2023.115047. 37536508

[ddr70198-bib-0090] Komura, H. , R. Watanabe , and K. Mizuguchi . 2023. “The Trends and Future Prospective of In Silico Models From the Viewpoint of ADME Evaluation in Drug Discovery.” Pharmaceutics 15, no. 11: 2619. 10.3390/PHARMACEUTICS15112619. 38004597 PMC10675155

[ddr70198-bib-0091] Kumar, A. , K. E. McNally , Y. Zhang , et al. 2025. “Multispectral Live‐Cell Imaging With Uncompromised Spatiotemporal Resolution.” Nature Photonics 19: 1146–1156. 10.1038/s41566-025-01745-7. 41048459 PMC12488498

[ddr70198-bib-0092] Kumar, V. , P. K. Chunchagatta Lakshman , T. K. Prasad , et al. 2024. “Target‐Based Drug Discovery: Applications of Fluorescence Techniques in High Throughput and Fragment‐Based Screening.” Heliyon 10, no. 1: e23864. 10.1016/j.heliyon.2023.e23864. 38226204 PMC10788520

[ddr70198-bib-0093] Lathram, W. A. , and C. D. Radka . 2025. “Intracellular Survival of *Staphylococcus aureus* in Macrophages During Osteomyelitis.” Virulence 16, no. 1: 2553789. 10.1080/21505594.2025.2553789. 40888369 PMC12408055

[ddr70198-bib-0094] Lazar, V. , E. Oprea , and L. M. Ditu . 2023. “Resistance, Tolerance, Virulence and Bacterial Pathogen Fitness.” Pathogens 12, no. 5: 746. 10.3390/pathogens12050746. 37242416 PMC10222143

[ddr70198-bib-0095] Le, C. F. , C. M. Fang , and S. D. Sekaran . 2017. “Intracellular Targeting Mechanisms by Antimicrobial Peptides.” Antimicrobial Agents and Chemotherapy 61, no. 4. 10.1128/AAC.02340-16. PMC5365711 28167546

[ddr70198-bib-0096] Lehar, S. M. , T. Pillow , M. Xu , et al. 2015. “Novel Antibody‐Antibiotic Conjugate Eliminates Intracellular *S. aureus* .” Nature 527, no. 7578: 323–328. 10.1038/NATURE16057;SUBJMETA. 26536114

[ddr70198-bib-0097] Leoni Swart, A. , and M. Hensel . 2012. “Interactions of *Salmonella enterica* With Dendritic Cells.” Virulence 3, no. 7: 660–667. 10.4161/VIRU.22761. 23221476 PMC3545948

[ddr70198-bib-0098] Li, J. , Y. Wei , C. Zhang , et al. 2023. “Cell‐Membrane‐Coated Nanoparticles for Targeted Drug Delivery to the Brain.” Pharmaceutics 15, no. 2: 621. 10.3390/pharmaceutics15020621. 36839943 PMC9960717

[ddr70198-bib-0099] Li, M. , K. G. Neoh , L. Xu , et al. 2016. “Sugar‐Grafted Cyclodextrin Nanocarrier as a ‘Trojan Horse’ for Potentiating Antibiotic Activity.” Pharmaceutical Research 33, no. 5: 1161–1174. 10.1007/s11095-016-1861-0. 26792570

[ddr70198-bib-0100] Li, Q. 2022. “Mechanisms for the Invasion and Dissemination of Salmonella.” Canadian Journal of Infectious Diseases and Medical Microbiology 2022: 2655801. 10.1155/2022/2655801. 35722038 PMC9203224

[ddr70198-bib-0101] Li, X. , S. Dong , Q. Pan , N. Liu , and Y. Zhang . 2025. “Antibiotic Conjugates: Using Molecular Trojan Horses to Overcome Drug Resistance.” Biomedicine & Pharmacotherapy 186: 118007. 10.1016/j.biopha.2025.118007. 40268370

[ddr70198-bib-0102] Lin, M. , A. Koley , W. Zhang , D. Pei , and Y. Rikihisa . 2023. “Inhibition of *Ehrlichia chaffeensis* Infection by Cell‐Permeable Macrocyclic Peptides That Bind Type IV Secretion Effector Etf‐1.” PNAS Nexus 2, no. 2: 017. 10.1093/pnasnexus/pgad017. PMC9982066 36874272

[ddr70198-bib-0103] Ling, X. , J. Tu , J. Wang , et al. 2019. “Glutathione‐Responsive Prodrug Nanoparticles for Effective Drug Delivery and Cancer Therapy.” ACS Nano 13, no. 1: 357–370. 10.1021/acsnano.8b06400. 30485068 PMC7049173

[ddr70198-bib-0104] Lipinski, C. A. 2004. “Lead‐ and Drug‐Like Compounds: The Rule‐of‐Five Revolution.” Drug Discovery Today: Technologies 1, no. 4: 337–341. 10.1016/j.ddtec.2004.11.007. 24981612

[ddr70198-bib-0105] Liu, X. , M. Li , and S. Woo . 2024. “Subcellular Drug Distribution: Exploring Organelle‐Specific Characteristics for Enhanced Therapeutic Efficacy.” Pharmaceutics 16, no. 9: 1167. 10.3390/PHARMACEUTICS16091167. 39339204 PMC11434838

[ddr70198-bib-0106] Liu, Y. , J. Luo , X. Chen , W. Liu , and T. Chen . 2019. “Cell Membrane Coating Technology: A Promising Strategy for Biomedical Applications.” Nano‐Micro Letters 11, no. 1: 100. 10.1007/s40820-019-0330-9. 34138027 PMC7770915

[ddr70198-bib-0107] Liu, Y. , K. Yang , H. Zhang , Y. Jia , and Z. Wang . 2020. “Combating Antibiotic Tolerance Through Activating Bacterial Metabolism.” Frontiers in Microbiology 11: 577564. 10.3389/fmicb.2020.577564. 33193198 PMC7642520

[ddr70198-bib-0108] Lu, K. Y. , X. Yang , M. J. G. Eldridge , et al. 2025. “A Host‐Directed Adjuvant Sensitizes Intracellular Bacterial Persisters to Antibiotics.” Nature Microbiology 10: 3013–3025. 10.1038/s41564-025-02124-2. PMC12578635 41073665

[ddr70198-bib-0109] Lu, M. , and Y. Huang . 2020. “Bioinspired Exosome‐Like Therapeutics and Delivery Nanoplatforms.” Biomaterials 242: 119925. 10.1016/j.biomaterials.2020.119925. 32151860

[ddr70198-bib-0110] Luan, X. , K. Sansanaphongpricha , I. Myers , H. Chen , H. Yuan , and D. Sun . 2017. “Engineering Exosomes as Refined Biological Nanoplatforms for Drug Delivery.” Acta Pharmacologica Sinica 38, no. 6: 754–763. 10.1038/APS.2017.12. 28392567 PMC5520184

[ddr70198-bib-0111] Luo, S. , Z. Lv , Q. Yang , R. Chang , and J. Wu . 2023. “Research Progress on Stimulus‐Responsive Polymer Nanocarriers for Cancer Treatment.” Pharmaceutics 15, no. 7: 1928. 10.3390/pharmaceutics15071928. 37514114 PMC10386740

[ddr70198-bib-0112] Ma, L. , Q. Ouyang , G. C. Werthmann , H. M. Thompson , and E. M. Morrow . 2017. “Live‐Cell Microscopy and Fluorescence‐Based Measurement of Luminal pH in Intracellular Organelles.” Frontiers in Cell and Developmental Biology 5: 71. 10.3389/fcell.2017.00071. 28871281 PMC5566985

[ddr70198-bib-0113] Mahieu, G. , L. Elens , N. Prebonnaud , A. Chauzy , and F. Van Bambeke . 2025. “A Hollow Fiber Infection Model to Study Intracellular and Extracellular Antibiotic Activity Against *Staphylococcus aureus* .” IScience 28, no. 3: 112076. 10.1016/j.isci.2025.112076. 40124509 PMC11930174

[ddr70198-bib-0114] Maphasa, R. E. , M. Meyer , and A. Dube . 2021. “The Macrophage Response to *Mycobacterium tuberculosis* and Opportunities for Autophagy‐Inducing Nanomedicines.” Frontiers in Cellular and Infection Microbiology 10: 618414. 10.3389/fcimb.2020.618414. 33628745 PMC7897680

[ddr70198-bib-0115] Marchi, J. , C. N. N. Minh , L. Debarbieux , and J. S. Weitz . 2025. “Multi‐Strain Phage Induced Clearance of Bacterial Infections.” PLoS Computational Biology 21, no. 2: e1012793. 10.1371/JOURNAL.PCBI.1012793. 39903766 PMC11828373

[ddr70198-bib-0116] Mateus, A. , A. Treyer , C. Wegler , M. Karlgren , P. Matsson , and P. Artursson . 2017. “Intracellular Drug Bioavailability: A New Predictor of System Dependent Drug Disposition.” Scientific Reports 7, no. 1: 43047. 10.1038/srep43047. 28225057 PMC5320532

[ddr70198-bib-0117] Matović, J. , J. Järvinen , H. C. Bland , et al. 2020. “Addressing the Biochemical Foundations of a Glucose‐Based ‘Trojan Horse’—Strategy.” Molecular Pharmaceutics 17, no. 10: 3885–3899. 10.1021/acs.molpharmaceut.0c00630. 32787269 PMC7539299

[ddr70198-bib-0118] McBride, J. W. , and D. H. Walker . 2011. “Molecular and Cellular Pathobiology of Ehrlichia Infection.” Expert Reviews in Molecular Medicine 13: e3. 10.1017/S1462399410001730. 21276277 PMC3767467

[ddr70198-bib-0119] Melander, R. J. , A. E. Mattingly , and C. Melander . 2022. “Phenotypic Screening of Compound Libraries as a Platform for Antibiotic Adjuvant Identification.” Methods in Enzymology 665: 153–176. 10.1016/bs.mie.2021.11.005. 35379433 PMC10942738

[ddr70198-bib-0120] Mishra, M. K. , K. Kotta , M. Hali , et al. 2011. “Pamam Dendrimer‐Azithromycin Conjugate Nanodevices for the Treatment of Chlamydia Trachomatis Infections.” Nanomedicine: Nanotechnology, Biology, and Medicine 7, no. 6: 935–944. 10.1016/j.nano.2011.04.008. 21658474

[ddr70198-bib-0121] Misra, B. B. , C. Langefeld , M. Olivier , and L. A. Cox . 2019. “Integrated Omics: Tools, Advances and Future Approaches.” Journal of Molecular Endocrinology 62, no. 1: R21–R45. 10.1530/JME-18-0055. 30006342

[ddr70198-bib-0122] Moreau, A. , M. Le Vee , E. Jouan , Y. Parmentier , and O. Fardel . 2011. “Drug Transporter Expression in Human Macrophages.” Fundamental & Clinical Pharmacology 25, no. 6: 743–752. 10.1111/j.1472-8206.2010.00913.x. 21210849

[ddr70198-bib-0123] Mugumbate, G. , and J. P. Overington . 2015. “The Relationship Between Target‐Class and the Physicochemical Properties of Antibacterial Drugs.” Bioorganic & Medicinal Chemistry 23, no. 16: 5218–5224. 10.1016/j.bmc.2015.04.063. 25975639 PMC4537081

[ddr70198-bib-0124] Mukherjee, A. , A. K. Waters , P. Kalyan , A. S. Achrol , S. Kesari , and V. M. Yenugonda . 2019. “Lipid–Polymer Hybrid Nanoparticles as a Next‐Generation Drug Delivery Platform: State of the Art, Emerging Technologies, and Perspectives.” International Journal of Nanomedicine 14: 1937–1952. 10.2147/IJN.S198353. 30936695 PMC6430183

[ddr70198-bib-0125] Mulcahy, M. E. , E. C. O'Brien , K. M. O'Keeffe , E. G. Vozza , N. Leddy , and R. M. McLoughlin . 2020. “Manipulation of Autophagy and Apoptosis Facilitates Intracellular Survival of *Staphylococcus aureus* in Human Neutrophils.” Frontiers in Immunology 11: 565545. 10.3389/FIMMU.2020.565545/BIBTEX. 33262756 PMC7686353

[ddr70198-bib-0126] Murray, S. M. , and P. F. McKay . 2021. “Chlamydia Trachomatis: Cell Biology, Immunology and Vaccination.” Vaccine 39, no. 22: 2965–2975. 10.1016/j.vaccine.2021.03.043. 33771390

[ddr70198-bib-0127] Narang, A. , R. K. Chang , and M. A. Hussain . 2013. “Pharmaceutical Development and Regulatory Considerations for Nanoparticles and Nanoparticulate Drug Delivery Systems.” Journal of Pharmaceutical Sciences 102, no. 11: 3867–3882. 10.1002/jps.23691. 24037829

[ddr70198-bib-0128] Necchi, V. , P. Sommi , A. Vanoli , R. Fiocca , V. Ricci , and E. Solcia . 2017. “Natural History of Helicobacter Pylori VacA Toxin in Human Gastric Epithelium In Vivo.” Scientific Reports 7: 14526. 10.1038/s41598-017-15204-z. 29109534 PMC5673961

[ddr70198-bib-0129] Ngwalero, P. , J. Brust , S. W. van Beek , et al. 2021. “Relationship Between Plasma and Intracellular Concentrations of Bedaquiline and Its M2 Metabolite in South African Patients With Rifampin‐Resistant Tuberculosis.” Antimicrobial Agents and Chemotherapy 65, no. 11: 0239920. 10.1128/AAC.02399-20. PMC8522761 34370588

[ddr70198-bib-0130] Niu, H. , Q. Xiong , A. Yamamoto , M. Hayashi‐Nishino , and Y. Rikihisa . 2012. “Autophagosomes Induced by a Bacterial Beclin 1 Binding Protein Facilitate Obligatory Intracellular Infection.” Proceedings of the National Academy of Sciences 109, no. 51: 20800–20807. 10.1073/pnas.1218674109. PMC3529060 23197835

[ddr70198-bib-0131] Noorain, L. , V. Nguyen , H. W. Kim , and L. T. B. Nguyen . 2023. “A Machine Learning Approach for PLGA Nanoparticles in Antiviral Drug Delivery.” Pharmaceutics 15, no. 2: 495. 36839817 10.3390/pharmaceutics15020495 PMC9966002

[ddr70198-bib-0132] Nowacki, J. S. , G. S. Jones , and S. E. F. D'Orazio . 2025. “ *Listeria monocytogenes* use Multiple Mechanisms to Disseminate From the Intestinal Lamina Propria to the Mesenteric Lymph Nodes.” Microbiology Spectrum 13 2: 0259524. 10.1128/spectrum.02595-24. PMC11792513 39714174

[ddr70198-bib-0133] Ocana, A. , A. Pandiella , C. Privat , et al. 2025. “Integrating Artificial Intelligence in Drug Discovery and Early Drug Development: A Transformative Approach.” Biomarker Research 13, no. 1: 45. 10.1186/S40364-025-00758-2. 40087789 PMC11909971

[ddr70198-bib-0134] Ogawa, M. , T. Yoshimori , T. Suzuki , H. Sagara , N. Mizushima , and C. Sasakawa . 2005. “Escape of Intracellular Shigella From Autophagy.” Science 307, no. 5710: 727–731. 10.1126/science.1106036. 15576571

[ddr70198-bib-0135] Ohashi, R. , R. Watanabe , T. Esaki , et al. 2019. “Development of Simplified In Vitro P‐Glycoprotein Substrate Assay and In Silico Prediction Models To Evaluate Transport Potential of P‐Glycoprotein.” Molecular Pharmaceutics 16, no. 5: 1851–1863. 10.1021/ACS.MOLPHARMACEUT.8B01143. 30933526

[ddr70198-bib-0136] Omotade, T. O. , and C. R. Roy . 2019. “Manipulation of Host Cell Organelles by Intracellular Pathogens.” Microbiology Spectrum 7, no. 2: 10–1128. 0022–2019. 10.1128/microbiolspec.BAI-0022-2019. PMC11590418 31025623

[ddr70198-bib-0137] Ozanic, M. , V. Marecic , Y. Abu Kwaik , and M. Santic . 2015. “The Divergent Intracellular Lifestyle of *Francisella tularensis* .” PLoS Pathogens 11, no. 12: 1005208. 10.1371/journal.ppat.1005208. PMC4669081 26633893

[ddr70198-bib-0138] Paredes‐Sabja, D. , A. Shen , and J. A. Sorg . 2014. “Clostridium Difficile Spore Biology: Sporulation, Germination, and Spore Structural Proteins.” Trends in Microbiology 22, no. 7: 406–416. 10.1016/j.tim.2014.04.003. 24814671 PMC4098856

[ddr70198-bib-0139] Patterson, L. L. , C. D. Byerly , R. Solomon , et al. 2023. “Ehrlichia notch Signaling Induction Promotes XIAP Stability and Inhibits Apoptosis.” Infection and Immunity 91, no. 9: 0000223. 10.1128/iai.00002-23. PMC10501217 37594275

[ddr70198-bib-0140] Pea, F. 2018. “Intracellular Pharmacokinetics of Antibacterials and Their Clinical Implications.” Clinical Pharmacokinetics 57, no. 2: 177–189. 10.1007/s40262-017-0572-y. 28639230

[ddr70198-bib-0141] Pea, F. , P. Viale , and M. Furlanut . 2005. “Antimicrobial Therapy in Critically Ill Patients.” Clinical Pharmacokinetics 44, no. 10: 1009–1034. 10.2165/00003088-200544100-00002. 16176116

[ddr70198-bib-0142] Pei, D. , and M. Buyanova . 2019. “Overcoming Endosomal Entrapment in Drug Delivery.” Bioconjugate Chemistry 30, no. 2: 273–283. 10.1021/acs.bioconjchem.8b00778. 30525488 PMC6501178

[ddr70198-bib-0143] Pei, X. M. , M. H. Y. Yeung , A. N. N. Wong , et al. 2023. “Targeted Sequencing Approach and Its Clinical Applications for the Molecular Diagnosis of Human Diseases.” Cells 12, no. 3: 493. 10.3390/cells12030493. 36766834 PMC9913990

[ddr70198-bib-0144] Perry, R. D. , and J. D. Fetherston . 1997. “Yersinia Pestis—Etiologic Agent of Plague.” Clinical Microbiology Reviews 10, no. 1: 35–66. 10.1128/CMR.10.1.35. 8993858 PMC172914

[ddr70198-bib-0145] Phat, V. V. , A. S. T. Lim , C. De cozar‐Gallardo , et al. 2025. “A Three‐Dimensional High Throughput Assay Identifies Novel Antibacterial Molecules With Activity Against Intracellular Shigella.” NPJ Antimicrobials and Resistance 3, no. 1: 40. 10.1038/s44259-025-00110-6. 40374850 PMC12081684

[ddr70198-bib-0146] Piccaro, G. , G. Poce , M. Biava , F. Giannoni , and L. Fattorini . 2015. “Activity of Lipophilic and Hydrophilic Drugs Against Dormant and Replicating *Mycobacterium tuberculosis* .” Journal of antibiotics 68, no. 11: 711–714. 10.1038/ja.2015.52. 25944535

[ddr70198-bib-0147] Pilla‐Moffett, D. , M. F. Barber , G. A. Taylor , and J. Coers . 2016. “Interferon‐Inducible GTPases in Host Resistance.” Journal of Molecular Biology 428, no. 17: 3495–3513. 10.1016/j.jmb.2016.04.032. 27181197 PMC5010443

[ddr70198-bib-0148] Pizarro‐Cerdá, J. , and P. Cossart . 2018. “ *Listeria monocytogenes*: Cell Biology of Invasion and Intracellular Growth.” Microbiology Spectrum 6, no. 10: 6. 10.1128/microbiolspec.gpp3-0013-2018. PMC11633638 30523778

[ddr70198-bib-0149] Prescott, J. F. 1991. “Rhodococcus equi: An Animal and Human Pathogen.” Clinical Microbiology Reviews 4, no. 1: 20–34. 10.1128/CMR.4.1.20. 2004346 PMC358176

[ddr70198-bib-0150] Pries, V. , S. Cotesta , R. Riedl , et al. 2016. “Advantages and Challenges of Phenotypic Screens.” SLAS Discovery 21, no. 3: 306–315. 10.1177/1087057115610488. 26459507

[ddr70198-bib-0151] Pryor, J. B. , B. J. Harper , and S. L. Harper . 2014. “Comparative Toxicological Assessment of PAMAM and Thiophosphoryl Dendrimers Using Embryonic Zebrafish.” International Journal of Nanomedicine 9, no. 1: 1947–1956. 10.2147/IJN.S60220. 24790436 PMC4000179

[ddr70198-bib-0152] Qiu, Y. , C. Lu , P. Chen , et al. 2019. “Synergistic Clearance of Intracellular Pathogens by Hyaluronan‐Streptomycin Micelles Encapsulated With Rapamycin.” Carbohydrate Polymers 210: 364–371. 10.1016/j.carbpol.2019.01.068. 30732772

[ddr70198-bib-0153] Quillin, S. J. , and H. S. Seifert . 2018. “Neisseria Gonorrhoeae Host Adaptation and Pathogenesis.” Nature Reviews Microbiology 16, no. 4: 226–240. 10.1038/nrmicro.2017.169. 29430011 PMC6329377

[ddr70198-bib-0154] Radolf, J. D. , R. K. Deka , A. Anand , D. Šmajs , M. V. Norgard , and X. F. Yang . 2016. “Treponema Pallidum, the Syphilis Spirochete: Making a Living as a Stealth Pathogen.” Nature Reviews Microbiology 14, no. 12: 744–759. 10.1038/nrmicro.2016.141. 27721440 PMC5106329

[ddr70198-bib-0155] Rajput, R. , J. Narkhede , and J. Naik . 2020. “Nanogels as Nanocarriers for Drug Delivery: A Review.” ADMET & DMPK 8, no. 1: 1–15. 10.5599/ADMET.724. 35299773 PMC8915596

[ddr70198-bib-0156] Rao, K. M. , M. Suneetha , D. V. Kumar , H. J. Kim , Y. J. Seok , and S. S. Han . 2022. “Dual Responsive Poly(Vinyl Caprolactam)‐Based Nanogels for Tunable Intracellular Doxorubicin Delivery in Cancer Cells.” Pharmaceutics 14, no. 4: 852. 10.3390/pharmaceutics14040852. 35456685 PMC9029372

[ddr70198-bib-0157] Rautio, J. , N. A. Meanwell , L. Di , and M. J. Hageman . 2018. “The Expanding Role of Prodrugs in Contemporary Drug Design and Development.” Nature Reviews Drug Discovery 17, no. 8: 559–587. 10.1038/nrd.2018.46. 29700501

[ddr70198-bib-0158] Rayner, B. , A. D. Verderosa , V. Ferro , and M. A. T. Blaskovich . 2023. “Siderophore Conjugates to Combat Antibiotic‐Resistant Bacteria.” RSC Medicinal Chemistry 14, no. 5: 800–822. 10.1039/D2MD00465H. 37252105 PMC10211321

[ddr70198-bib-0159] Richards, A. L. , and J. Jiang . 2020. “Scrub Typhus: Historic Perspective and Current Status.” Tropical Medicine and Infectious Disease 5, no. 2: 49. 10.3390/tropicalmed5020049. 32244598 PMC7344502

[ddr70198-bib-0160] Salam, M. A. , M. Y. Al‐Amin , M. T. Salam , et al. 2023. “Antimicrobial Resistance: A Growing Serious Threat for Global Public Health.” Healthcare 11, no. 13: 1946. 10.3390/healthcare11131946. 37444780 PMC10340576

[ddr70198-bib-0161] Sánchez, A. , S. P. Mejía , and J. Orozco . 2020. “Recent Advances in Polymeric Nanoparticle‐Encapsulated Drugs Against Intracellular Infections.” Molecules 25, no. 16: 3760. 10.3390/MOLECULES25163760. 32824757 PMC7464666

[ddr70198-bib-0162] Sankar, P. , and B. B. Mishra . 2023. “Early Innate Cell Interactions With *Mycobacterium tuberculosis* .” Frontiers in Immunology 14: 1260859. 10.3389/fimmu.2023.1260859. 37965344 PMC10641450

[ddr70198-bib-0163] Schmalstig, A. A. , A. Wiggins , D. Badillo , K. S. Wetzel , G. F. Hatfull , and M. Braunstein . 2024. “Bacteriophage Infection and Killing of Intracellular *Mycobacterium abscessus* .” mBio 15, no. 1. 10.1128/MBIO.02924-23. PMC10790704 38059609

[ddr70198-bib-0164] Schnupf, P. , and P. J. Sansonetti . 2019. “Shigella Pathogenesis: New Insights Through Advanced Methodologies.” Microbiology Spectrum 7, no. 2: 10–1128. 0023–2019. 10.1128/microbiolspec.BAI-0023-2019. PMC11588159 30953429

[ddr70198-bib-0165] Shah, S. , P. Famta , R. S. Raghuvanshi , S. B. Singh , and S. Srivastava . 2022. “Lipid Polymer Hybrid Nanocarriers: Insights Into Synthesis Aspects, Characterization, Release Mechanisms, Surface Functionalization and Potential Implications.” Colloid and Interface Science Communications 46: 100570. 10.1016/J.COLCOM.2021.100570.

[ddr70198-bib-0166] Sharma, A. , V. K. Gupta , and R. Pathania . 2019. “Efflux Pump Inhibitors for Bacterial Pathogens.” Indian Journal of Medical Research 149, no. 2: 129–145. 10.4103/ijmr.IJMR_2079_17. 31219077 PMC6563736

[ddr70198-bib-0167] Sharma, A. , and G. Khuller . 2001. “DNA Vaccines: Future Strategies.” Immunology & Cell Biology 79, no. 6: 537–546. 10.1046/j.1440-1711.2001.01044.x. 11903613

[ddr70198-bib-0168] Simkhada, D. , S. H. C. Teo , N. Deorkar , and M. C. Vemuri . 2025. “Transfection Technologies for Next‐Generation Therapies.” Journal of Clinical Medicine 14, no. 15: 5515. 10.3390/jcm14155515. 40807133 PMC12347970

[ddr70198-bib-0169] Simplício, A. L. , J. M. Clancy , and J. F. Gilmer . 2007. “β‐Aminoketones as Prodrugs With pH‐Controlled Activation.” International Journal of Pharmaceutics 336, no. 2: 208–214. 10.1016/j.ijpharm.2006.11.055. 17197138

[ddr70198-bib-0170] Song, J. , X. Wu , Y. Kong , et al. 2022. “Prevalence and Antibiotic Resistance of Ureaplasma Species.” Frontiers in Microbiology 13: 982429. 10.3389/fmicb.2022.982429. 36187990 PMC9520197

[ddr70198-bib-0171] Song, Y. , D. Li , J. He , M. Zhang , and P. Ni . 2019. “Facile Preparation of pH‐Responsive Pegylated Prodrugs.” Chinese Chemical Letters 30, no. 12: 2027–2031. 10.1016/j.cclet.2019.04.052.

[ddr70198-bib-0172] Steele‐Mortimer, O. 2008. “The Salmonella‐Containing Vacuole: Moving With the Times.” Current Opinion in Microbiology 11, no. 1: 38–45. 10.1016/j.mib.2008.01.002. 18304858 PMC2577838

[ddr70198-bib-0173] Stephens, F. B. , D. Constantin‐Teodosiu , and P. L. Greenhaff . 2007. “New Insights Concerning the Role of Carnitine in the Regulation of Fuel Metabolism in Skeletal Muscle.” Journal of Physiology 581, no. Pt 2: 431–444. 10.1113/jphysiol.2006.125799. 17331998 PMC2075186

[ddr70198-bib-0174] Stokes, J. M. , A. J. Lopatkin , M. A. Lobritz , and J. J. Collins . 2019. “Bacterial Metabolism and Antibiotic Efficacy.” Cell Metabolism 30, no. 2: 251–259. 10.1016/j.cmet.2019.06.009. 31279676 PMC6990394

[ddr70198-bib-0175] Subramaniam, S. , P. Joyce , C. E. Conn , and C. A. Prestidge . 2024. “Cellular Uptake Andin Vitroantibacterial Activity of Lipid‐Based Nanoantibiotics Are Influenced by Protein Corona.” Biomaterials Science 12, no. 13: 3411–3422. 10.1039/D4BM00608A. 38809118

[ddr70198-bib-0176] Subramaniam, S. , P. Joyce , N. Thomas , and C. A. Prestidge . 2021. “Bioinspired Drug Delivery Strategies for Repurposing Conventional Antibiotics Against Intracellular Infections.” Advanced Drug Delivery Reviews 177: 113948. 10.1016/j.addr.2021.113948. 34464665

[ddr70198-bib-0177] Subramanian, I. , S. Verma , S. Kumar , A. Jere , and K. Anamika . 2019. “Multi‐Omics Data Integration, Interpretation, and Its Application.” Bioinformatics and biology insights 14: 1177932219899051. 10.1177/1177932219899051 PMC7003173 32076369

[ddr70198-bib-0178] Subramanian, I. , S. Verma , S. Kumar , A. Jere , and K. Anamika . 2020. “Multi‐Omics Data Integration, Interpretation, and Its Application.” Bioinformatics and biology insights 14: 117793221989905. 10.1177/1177932219899051. PMC7003173 32076369

[ddr70198-bib-0179] Syed, I. , and R. M. Wooten . 2021. “Interactions Between Pathogenic Burkholderia and the Complement System.” Frontiers in Cellular and Infection Microbiology 11: 701362. 10.3389/fcimb.2021.701362. 34660335 PMC8515183

[ddr70198-bib-0180] Tang, Q. , P. Tan , Z. Dai , et al. 2023. “Hydrophobic Modification Improves the Delivery of Cell‐Penetrating Peptides.” Acta Biomaterialia 157: 210–224. 10.1016/j.actbio.2022.11.055. 36503077

[ddr70198-bib-0181] Tantama, M. , Y. P. Hung , and G. Yellen . 2011. “Imaging Intracellular pH in Live Cells With a Genetically Encoded Red Fluorescent Protein Sensor.” Journal of the American Chemical Society 133, no. 26: 10034–10037. 10.1021/ja202902d. 21631110 PMC3126897

[ddr70198-bib-0182] Tao, J. , Y. Gu , W. Zhou , and Y. Wang . 2025. “Dual‐Payload Antibody‐Drug Conjugates: Taking a Dual Shot.” European Journal of Medicinal Chemistry 281: 116995. 10.1016/j.ejmech.2024.116995. 39481229

[ddr70198-bib-0183] Testa, B. 2009. “Prodrugs: Bridging Pharmacodynamic/Pharmacokinetic Gaps.” Current Opinion in Chemical Biology 13, no. 3: 338–344. 10.1016/j.cbpa.2009.04.620. 19473869

[ddr70198-bib-0184] Thakur, A. , H. Mikkelsen , and G. Jungersen . 2019. “Intracellular Pathogens: Host Immunity and Microbial Persistence Strategies.” Journal of Immunology Research 2019: 1356540. 10.1155/2019/1356540. 31111075 PMC6487120

[ddr70198-bib-0185] Thomas, M. , A. Boardman , M. Garcia‐Ortegon , H. Yang , C. de Graaf , and A. Bender . 2022. “Applications of Artificial Intelligence in Drug Design: Opportunities and Challenges.” Methods in Molecular Biology 2390: 1–59. 10.1007/978-1-0716-1787-8_1. 34731463

[ddr70198-bib-0186] Thy, M. , J.‐F. Timsit , and E. de Montmollin . 2023. “Aminoglycosides for the Treatment of Severe Infection Due to Resistant Gram‐Negative Pathogens.” Antibiotics (USSR) 12, no. 5: 860. 10.3390/antibiotics12050860. PMC10215420 37237763

[ddr70198-bib-0187] Tian, J. H. , S. Huang , Z. H. Wang , et al. 2025. “Supramolecular Discrimination and Diagnosis‐Guided Treatment of Intracellular Bacteria.” Nature Communications 16: 1016. 10.1038/s41467-025-56308-9. PMC11762306 39863571

[ddr70198-bib-0188] Tian, N. , H. Chu , Q. Li , et al. 2025. “Host‐Directed Therapy for Tuberculosis.” European Journal of Medical Research 30: 267. 10.1186/s40001-025-02443-4. 40211397 PMC11987284

[ddr70198-bib-0189] Tripathi, D. , P. Pandey , S. Sharma , et al. 2025. “Advances in Nanomaterials for Precision Drug Delivery.” BioImpacts: BI 15: 30573. 10.34172/bi.30573. 40256227 PMC12008503

[ddr70198-bib-0190] Truchan, H. K. , C. L. Cockburn , K. S. Hebert , F. Magunda , S. M. Noh , and J. A. Carlyon . 2016. “The Pathogen‐Occupied Vacuoles of *Anaplasma phagocytophilum* and *Anaplasma marginale* Interact With the Endoplasmic Reticulum.” Frontiers in Cellular and Infection Microbiology 6, no. MAR: 22. 10.3389/FCIMB.2016.00022. 26973816 PMC4771727

[ddr70198-bib-0191] Turck, J. W. , H. Sultana , and G. Neelakanta . 2025. “Arthropod Autophagy Molecules Facilitate *Anaplasma phagocytophilum* Infection of Ixodes Scapularis.” Communications Biology 8: 433. 10.1038/s42003-025-07859-6. 40082564 PMC11906822

[ddr70198-bib-0192] Upadhyay, T. K. , N. Fatima , D. Sharma , V. Saravanakumar , and R. Sharma . 2017. “Preparation and Characterization of Beta‐Glucan Particles Containing a Payload of Nanoembedded Rifabutin for Enhanced Targeted Delivery to Macrophages.” EXCLI Journal 16: 210–228. 10.17179/EXCLI2016-804. 28507467 PMC5427468

[ddr70198-bib-0193] Valbuena, G. , and D. H. Walker . 2009. “Infection of the Endothelium by Members of the Order Rickettsiales.” Thrombosis and Haemostasis 102, no. 6: 1071–1079. 10.1160/TH09-03-0186/ID/JR0186-6/BIB. 19967137 PMC2913309

[ddr70198-bib-0194] van Schaik, E. J. , A. P. Fratzke , A. E. Gregory , J. E. Dumaine , and J. E. Samuel . 2024. “Vaccine Development: Obligate Intracellular Bacteria—New Tools, Old Pathogens.” Frontiers in Cellular and Infection Microbiology 14: 1282183. 10.3389/fcimb.2024.1282183. 38567021 PMC10985213

[ddr70198-bib-0195] Vasaikar, S. , P. Bhatia , P. Bhatia , and K. Chu Yaiw . 2016. “Complementary Approaches to Existing Target‐Based Drug Discovery.” Biomedicines 4, no. 4: 27. 10.3390/biomedicines4040027. 28536394 PMC5344266

[ddr70198-bib-0196] Vashist, A. , G. Perez Alvarez , V. Andion Camargo , et al. 2024. “Recent Advances in Nanogels for Drug Delivery and Biomedical Applications.” Biomaterials Science 12, no. 23: 6006–6018. 10.1039/D4BM00224E. 39484856 PMC11528912

[ddr70198-bib-0197] Vergalli, J. , A. Atzori , J. Pajovic , et al. 2020. “The Challenge of Intracellular Antibiotic Accumulation, a Function of Fluoroquinolone Influx Versus Bacterial Efflux.” Communications Biology 3, no. 1: 198. 10.1038/s42003-020-0929-x. 32346058 PMC7189378

[ddr70198-bib-0198] Verkhovskii, R. , A. Ivanov , E. Lengert , K. Tulyakova , N. Shilyagina , and A. Ermakov . 2023. “Current Principles, Challenges, and New Metrics in pH‐Responsive Drug Delivery Systems for Systemic Cancer Therapy.” Pharmaceutics 15, no. 5: 1566. 10.3390/pharmaceutics15051566. 37242807 PMC10222897

[ddr70198-bib-0199] Vincent, F. , A. Nueda , J. Lee , M. Schenone , M. Prunotto , and M. Mercola . 2022. “Phenotypic Drug Discovery: Recent Successes, Lessons Learned and New Directions.” Nature Reviews Drug Discovery 21, no. 12: 899–914. 10.1038/s41573-022-00472-w. 35637317 PMC9708951

[ddr70198-bib-0200] Vora, L. K. , A. D. Gholap , K. Jetha , R. R. S. Thakur , H. K. Solanki , and V. P. Chavda . 2023. “Artificial Intelligence in Pharmaceutical Technology and Drug Delivery Design.” Pharmaceutics 15, no. 7: 1916. 10.3390/PHARMACEUTICS15071916. 37514102 PMC10385763

[ddr70198-bib-0301] Wan, W. , S. Zhang , M. Zhao , et al. 2024. “Lysosomal Trafficking Regulator Restricts Intracellular Growth of Coxiella burnetii by Inhibiting the Expansion of Coxiella‐Containing Vacuole and Upregulating nos2 Expression.” Frontiers in Cellular and Infection Microbiology 13: 1336600. 10.3389/fcimb.2023.1336600. 38282619 PMC10812120

[ddr70198-bib-0201] Wang, C. , Y. Yang , Y. Cao , et al. 2023. “Nanocarriers for the Delivery of Antibiotics Into Cells Against Intracellular Bacterial Infection.” Biomaterials Science 11, no. 2: 432–444. 10.1039/D2BM01489K. 36503914

[ddr70198-bib-0202] Wang, Q. , Y. Liu , C. Li , B. Xu , S. Xu , and B. Liu . 2025. “Machine Learning‐Enhanced Nanoparticle Design for Precision Cancer Drug Delivery.” Advanced Science 12, no. 30. 10.1002/ADVS.202503138. PMC12376635 40536233

[ddr70198-bib-0203] Wang, Y. , H. Li , A. Rasool , H. Wang , R. Manzoor , and G. Zhang . 2024. “Polymeric Nanoparticles (PNPs) for Oral Delivery of Insulin.” Journal of Nanobiotechnology 22, no. 1: 1–31. 10.1186/S12951-024-02576-4/FIGURES/7. 38167129 PMC10763344

[ddr70198-bib-0204] Wardecki, D. , M. Dołowy , and K. Bober‐Majnusz . 2023. “Assessment of Lipophilicity Parameters of Antimicrobial and Immunosuppressive Compounds.” Molecules 28, no. 6: 2820. 10.3390/molecules28062820. 36985792 PMC10059999

[ddr70198-bib-0205] Weng, Y. , H. Chen , X. Chen , H. Yang , C.‐H. Chen , and H. Tan . 2022. “Adenosine Triphosphate‐Activated Prodrug System for On‐Demand Bacterial Inactivation.” Nature Communications 13, no. 1: 4712. 10.1038/s41467-022-32453-3. PMC9372092 35953495

[ddr70198-bib-0206] Werren, J. H. , L. Baldo , and M. E. Clark . 2008. “Wolbachia: Master Manipulators of Invertebrate Biology.” Nature Reviews Microbiology 6, no. 10: 741–751. 10.1038/nrmicro1969. 18794912

[ddr70198-bib-0207] Winkle, M. , S. M. El‐Daly , M. Fabbri , and G. A. Calin . 2021. “Noncoding RNAs in Immune Regulation and Disease.” Nature Reviews Immunology 21, no. 9: 542–557. 10.1038/s41577-021-00566-8.

[ddr70198-bib-0208] Winnicka, K. , M. Wroblewska , P. Wieczorek , P. Sacha , and E. Tryniszewska . 2013. “The Effect of PAMAM Dendrimers on the Antibacterial Activity of Antibiotics With Different Water Solubility.” Molecules 18, no. 7: 8607–8617. 10.3390/MOLECULES18078607. 23881050 PMC6269725

[ddr70198-bib-0209] Wrońska, N. , A. Felczak , K. Zawadzka , et al. 2015. “Poly(Propylene Imine) Dendrimers and Amoxicillin as Dual‐Action Antibacterial Agents.” Molecules 20, no. 10: 19330–19342. 10.3390/molecules201019330. 26512634 PMC6331957

[ddr70198-bib-0210] Xavier, J. A. , T. L. Silva , E. C. Torres‐Santos , et al. 2021. “Redox Character of Nitroaromatic Compounds as Potential Medicines.” Curr Opin Electrochem 29: 100740. 10.1016/j.coelec.2021.100740.

[ddr70198-bib-0212] Xue, R. , M. Xie , Z. Wu , et al. 2024. “Mesenchymal Stem Cell‐Derived Exosomes Promote Recovery of The Facial Nerve Injury Through Regulating Macrophage M1 and M2 Polarization by Targeting the P38 MAPK/NF‐Κb Pathway.” Aging and Disease 15, no. 2: 851. 10.14336/AD.2023.0719-1. 37548941 PMC10917525

[ddr70198-bib-0213] Yang, J. , A. des Rieux , and A. Malfanti . 2025. “Stimuli‐Responsive Nanomedicines for the Treatment of Non‐Cancer Related Inflammatory Diseases.” ACS Nano 19, no. 16: 15189–15219. 10.1021/acsnano.5c00700. 40249331 PMC12045021

[ddr70198-bib-0214] Yang, L. , Y. Sun , Y. Xu , et al. 2020. “Antibacterial Peptide BSN‐37 Kills Extra‐ and Intra‐Cellular *Salmonella enterica* Serovar Typhimurium by a Nonlytic Mode of Action.” Frontiers in Microbiology 11: 74. 10.3389/FMICB.2020.00174. 32117178 PMC7019029

[ddr70198-bib-0215] Yousef, M. , T. S. Le , J. Zuo , et al. 2023. “Sub‐Cellular Sequestration of Alkaline Drugs in Lysosomes.” Research in Pharmaceutical Sciences 18, no. 1: 1–15. 10.4103/1735-5362.363591. 36846734 PMC9951787

[ddr70198-bib-0216] Zahra, M. J. , H. Hamed , R. Y. Mohammad , Z. Nosratollah , A. Akbarzadeh , and M. Morteza . 2017. “Evaluation and Study of Antimicrobial Activity of Nanoliposomal Meropenem Against Pseudomonas Aeruginosa Isolates.” Artificial Cells, Nanomedicine, and Biotechnology 45, no. 5: 975–980. 10.1080/21691401.2016.1198362. 27322561

[ddr70198-bib-0217] Zeballos, C. , M. A., and T. Gaj . 2021. “Next‐Generation CRISPR Technologies and Their Applications in Gene and Cell Therapy.” Trends in Biotechnology 39, no. 7: 692–705. 10.1016/j.tibtech.2020.10.010. 33277043 PMC8166939

[ddr70198-bib-0218] Zhang, D. , L. Yu , H. Tang , and H. Niu . 2025. “ *Anaplasma phagocytophilum* AFAP Targets the Host Nucleolus.” Frontiers in Microbiology 15: 1533640. 10.3389/fmicb.2024.1533640. 39839117 PMC11747512

[ddr70198-bib-0219] Zhang, L. , H. Xie , Y. Wang , H. Wang , J. Hu , and G. Zhang . 2022. “PK/PD Integration Models for Pharmacodynamics.” Frontiers in Veterinary Science 9: 860472. 10.3389/fvets.2022.860472. 35400105 PMC8989418

[ddr70198-bib-0220] Zhang, Y. , J. Zhang , W. Chen , et al. 2017. “Erythrocyte Membrane‐Coated Nanogel for Combinatorial Antivirulence and Responsive Antimicrobial Delivery Against *Staphylococcus aureus* Infection.” Journal of Controlled Release 263: 185–191. 10.1016/j.jconrel.2017.01.016. 28087406 PMC5503807

[ddr70198-bib-0221] Zhao, M. , X. Tan , Z. Liu , et al. 2023. “Engineered Phage With Cell‐Penetrating Peptides for Intracellular Bacterial Infections.” mSystems 8, no. 5. 10.1128/MSYSTEMS.00646-23. PMC10654057 37594262

[ddr70198-bib-0222] Zhao, M. , S. Zhang , W. Wan , et al. 2024. “Coxiella Burnetii Effector Cvpe Maintains Vacuole Biogenesis.” Virulence 15, no. 1. 10.1080/21505594.2024.2350893. PMC11085968 38725096

[ddr70198-bib-0223] Zhao, Y. , R. Zhong , Y. Fu , Z. Zhou , M. Yang , and L. He . 2021. “Simple and Feasible Design of a Polymeric Nanoparticle for Efficient Anticancer Drug Delivery.” Chemical Papers 75, no. 8: 4035–4044. 10.1007/S11696-021-01589-9/FIGURES/6.

[ddr70198-bib-0224] Zhong, G. , X. Chang , W. Xie , and X. Zhou . 2024. “Targeted Protein Degradation: Advances in Drug Discovery and Clinical Practice.” Signal Transduction and Targeted Therapy 9: 308. 10.1038/s41392-024-02004-x. 39500878 PMC11539257

[ddr70198-bib-0225] Zhou, X. , Y. Wu , Z. Zhu , et al. 2025. “Mucosal Immune Response in Biology, Disease Prevention and Treatment.” Signal Transduction and Targeted Therapy 10: 7. 10.1038/s41392-024-02043-4. 39774607 PMC11707400

[ddr70198-bib-0226] Zhu, C. , J. Mu , and L. Liang . 2024. “Nanocarriers for Intracellular Delivery of Proteins in Biomedical Applications: Strategies and Recent Advances.” Journal of Nanobiotechnology 22, no. 1: 688. 10.1186/s12951-024-02969-5. 39523313 PMC11552240

